# Role of Hepatitis B virus capsid phosphorylation in nucleocapsid disassembly and covalently closed circular DNA formation

**DOI:** 10.1371/journal.ppat.1008459

**Published:** 2020-03-30

**Authors:** Jun Luo, Ji Xi, Lu Gao, Jianming Hu

**Affiliations:** 1 Department of Microbiology and Immunology, The Pennsylvania State University College of Medicine, Hershey, Pennsylvania, United States of America; 2 Roche Pharma Research and Early Development, Roche Innovation Center Shanghai, Shanghai, China; University of California, San Diego, UNITED STATES

## Abstract

Hepatitis B virus (HBV) delivers a partially double-stranded, relaxed circular (RC) DNA genome in complete virions to the host cell nucleus for conversion to the covalently closed circular (CCC) DNA, which establishes and sustains viral infection. An overlength pregenomic RNA (pgRNA) is then transcribed from CCC DNA and packaged into immature nucleocapsids (NCs) by the viral core (HBc) protein. pgRNA is reverse transcribed to produce RC DNA in mature NCs, which are then enveloped and secreted as complete virions, or delivered to the nucleus to replenish the nuclear CCC DNA pool. RC DNA, whether originating from extracellular virions or intracellular mature NCs, must be released upon NC disassembly (uncoating) for CCC DNA formation. HBc is known to undergo dynamic phosphorylation and dephosphorylation at its C-terminal domain (CTD) to facilitate pgRNA packaging and reverse transcription. Here, two putative phosphorylation sites in the HBc N-terminal domain (NTD), S44 and S49, were targeted for genetic and biochemical analysis to assess their potential roles in viral replication. The NTD mutant that mimics the non-phosphorylated state (N2A) was competent in all steps of viral replication tested from capsid assembly, pgRNA packaging, reverse transcription, to virion secretion, except for a decrease in CCC DNA formation. On the other hand, the phosphor-mimetic mutant N2E showed a defect in the early step of pgRNA packaging but enhanced the late step of mature NC uncoating and consequently, increased CCC DNA formation. N2E also enhanced phosphorylation in CTD and possibly elsewhere in HBc. Furthermore, inhibition of the cyclin-dependent kinase 2 (CDK2), which is packaged into viral capsids, could block CCC DNA formation. These results prompted us to propose a model whereby rephosphorylation of HBc at both NTD and CTD by the packaged CDK2, following CTD dephosphorylation during NC maturation, facilitates uncoating and CCC DNA formation by destabilizing mature NCs.

## Author summary

Hepatitis B virus (HBV) persistently infects hundreds of millions of people worldwide, causing viral hepatitis, cirrhosis and liver cancer. The basis of HBV persistence is the viral covalently closed circular (CCC) DNA, a nuclear episome, that drives all viral gene expression to sustain viral replication. CCC DNA is derived from the relaxed circular (RC) DNA, which is formed inside a proteinaceous shell, the viral capsid, but has to be released from the capsid in order to be converted to CCC DNA by host cell factors. We report here that the phosphorylation state of the capsid protein, regulated by host cell enzymes including one that is packaged inside the viral capsid, plays a critical role in regulating the release of RC DNA and thus controlling CCC DNA formation. Intense ongoing efforts are being directed at developing novel antiviral strategies to eliminate the HBV CCC DNA for curing persistence HBV infection, including those targeting the capsid protein. Our results should inspire novel approaches to targeting the HBV capsid and CCC DNA. Furthermore, uncoating is an essential step in the infection process for virtually all viruses that remains ill-understood. Thus, our results have broad implications for understanding viral infection in general.

## Introduction

Hepatitis B virus (HBV), the prototype member of *Hepadnaviridae* family, is a major cause of viral hepatitis, liver cirrhosis, and hepatocellular carcinoma worldwide [1]. HBV has a small (ca. 3.2 kbp), partially double-stranded (DS), relaxed circular (RC) DNA genome [2] which is replicated via reverse transcription from an RNA intermediate, the pregenomic RNA (pgRNA) [3, 4]. The RC DNA genome is packaged into an icosahedral capsid composed of the HBV core protein (HBc), which in turn is enclosed in an envelope layer composed of host cell derived lipid bilayer studded with three viral envelope or surface proteins [5].

Upon infection of susceptible host cells, the HBV RC DNA genome is delivered to the nucleus where it is repaired to form the covalently closed circular (CCC) DNA [6, 7]. CCC DNA then serves as the viral transcriptional template to produce all viral RNAs, including pgRNA, required for replication. In addition to serving as the template for reverse transcription to replicate the viral DNA genome, pgRNA also serves as the mRNA for translating both HBc and the viral reverse transcriptase (RT) protein. Progeny virus assembly starts with the formation of a replication-competent nucleocapsid (NC) whereby the assembling HBc subunits (as homodimers) incorporate a single copy each of the RT protein and pgRNA, as a ribonucleoprotein (RNP) complex. Viral DNA synthesis ensues in the resulting pgRNA-containing NC to produce first the single-stranded (SS), minus strand DNA and then the RC DNA. NCs containing pgRNA or SS DNA are considered immature as they are not secreted outside the cells as virions; only RC DNA-containing (mature) NCs are competent for interaction with the viral envelope proteins and secreted extracellularly as complete virions [5]. Interestingly, the newly formed progeny RC DNA in intracellular mature NCs, like that from the incoming virion, can also be delivered to the host cell nucleus to form more CCC DNA [8–12]. This so-called intracellular CCC DNA amplification pathway effectively increases the HBV CCC DNA pool in the nucleus and is thought to be a critical feature of HBV replication for sustaining viral persistence [7, 13, 14].

HBc is a small (ca. 21 kDa) protein that plays multiple roles in HBV replication in addition to forming the shell of the NC [15]. It contains a globular N-terminal domain (NTD) and a C-terminal domain (CTD). The HBc NTD (amino acid 1–140), also known as the assembly domain, is essential for capsid formation [16, 17], but also plays a role in pgRNA packaging and reverse transcription [18]. The HBc CTD (amino acid 150–183) shows non-specific nucleic acid-binding activity and is essential for pgRNA packaging and reverse transcription [19–21]. In addition, the short (9-residues, 141–149) linker peptide between the NTD and CTD, traditionally thought to be simply a flexible spacer between the two domains, has been shown recently to play critical roles during multiple stages of viral replication [22].

With respect to the role of HBc in CCC DNA formation, current evidence suggests that HBc can control CCC DNA formation via at least two separate mechanisms. First, delivery of the RC DNA into the nucleus for conversion to CCC DNA is controlled by nuclear localization signals (NLSs) contained in the HBc CTD [23–25]. Also, mutations in the NTD such as L95A and K96A have been shown to increase CCC DNA formation, possibly also through their potential effects on nuclear import of RC DNA [26]. Second, HBc controls uncoating or disassembly of mature NCs to release RC DNA for CCC DNA formation [26, 27], with the generation of protein free (PF)-RC DNA, in which the RT protein covalently attached to the 5’ end of the minus strand of RC DNA has been removed, being an indication of (at least partial) uncoating [10]. PF-RC DNA may in fact be a mixture of multiple species each with a slightly different fine structure, and it remains uncertain if any of these are true intermediates in CCC DNA formation from RC DNA [10, 11, 28]. On the other hand, one particular form of PF-RC DNA, the closed minus strand RC DNA or cM-RC DNA, in which the minus strand of RC DNA is covalently closed but the plus strand remains open, represent the most likely intermediate to date in RC DNA to CCC DNA conversion [29].

The HBc CTD is known to undergo dynamic phosphorylation and dephosphorylation, which regulate HBc functions in capsid assembly [30] as well as in pgRNA packaging and reverse transcription [31–44]. Three major sites, and four minor sites of CTD phosphorylation, have been identified [23, 32, 36, 45]. The host cell cyclin-dependent kinase 2 (CDK2) and Polo-like kinase 1 (PLK-1) have been shown to phosphorylate the serine-proline (SP) and non-SP sites, respectively [45, 46] although additional kinases such as protein kinase C and the serine-arginine protein kinase have also been reported [34, 47–50]. Furthermore, a cellular kinase(s) is well known to be packaged into HBV capsids (the so-called endogenous kinase), and recent evidence indicates that the cyclin-dependent kinase 2 (CDK2) represents the major endogenous kinase, at least for capsids isolated from cultured human hepatoma cells [45].

In contrast to extensive studies on the role of CTD phosphorylation in HBV replication, little is known about potential phosphorylation of HBc NTD and its role in viral replication. We therefore decided to target two highly conserved and putative sites of phosphorylation in the HBc NTD. Our results obtained with HBc mutants that either block phosphorylation or mimic constitutive phosphorylation at these two NTD sites are consistent with a role of transient NTD phosphorylation, following NC maturation, in destabilizing the mature NC and facilitating its disassembly. Furthermore, our results suggest that phosphorylation at these HBc NTD sites can enhance phosphorylation at the CTD and possibly elsewhere in HBc to facilitate uncoating, and the endogenous kinase may play a major role in mediating these phosphorylation events during capsid uncoating.

## Materials and methods

### Ethics statement

Human primary hepatocytes from human liver chimeric mice (PXB cell) was purchased commercially from PhoenixBio (Hiroshima, Japan).

### Cell cultures and plasmids

HepG2, HEK293T and Huh7 cells were maintained in Dulbecco modified Eagle F-12 (DMEM-F12) medium supplemented with 10% fetal bovine serum, 50 ug/ml of penicillin-streptomycin. The HepAD38 cells were maintained in DMEM-F12 medium supplemented with 10% fetal bovine serum (FBS), 50 ug/ml of penicillin-streptomycin, 400 ug/ml G418, and 5 ug/ml of tetracycline (Tet). AD38 cells were induced to express HBV pgRNA upon removal of Tet from the culture medium [51]. The HepG2-NTCP cell line [29] was derived from the human hepatoma cell line HepG2 and stably expresses the HBV receptor, sodium taurocholate cotransporting polypeptide (NTCP). HepG2-NTCP cells were maintained in DMEM-F12 supplemented with 10% FBS and 50 ug/ml of penicillin-streptomycin. pCI-HBc WT, pCI-HBc-3A, pCI-HBc-3E pCI-HBc-7A, pCI-HBc-7E have been previously described [30]. pCI-HBc-N2A and pCI-HBc-N2E were constructed by changing two NTD putative phosphorylation sites (S44 and S49) to A and E, respectively, via PCR-mediated mutagenesis. pCI-HBc-9A and pCI-HBc-9E were constructed by changing all nine phosphorylation sites (S44 and S49 from NTD and all 7 S/T from CTD) to A and E, respectively. The HBV replicon plasmid used here, pCIΔA-HBV-HBc-WT, was constructed by subcloning the entire HBV insert together with the linked human cytomegalovirus (HCMV) promoter sequences from pCMVHBV [52, 53] to the pCIΔA vector [26]. pCIΔA-HBV-N2A and pCIΔA-HBV-N2E were constructed by subcloning the N2A and N2E mutations into pCIΔA-HBV-HBc-WT and express the mutated HBV pgRNA encoding the same HBc NTD mutations as in pCI-HBc-N2A and pCI-HBc-N2E, respectively.

### Antibody

The mouse monoclonal antibody (mAb) clone T2221 against the HBc NTD was purchased from Tokyo Future Style (Cat no. 2AHC24) [22]. The HBc CTD-specific rabbit mAb 25–7 and 6–1 were custom-made (by Abcam) using a non-phosphorylated CTD peptide (from residue 152 to the C terminus) as an immunogen and have been described before [30]. A701 is a mouse mAb prepared using a non-phosphorylated HBc CTD peptide from position 156 to 176 as the immunogen and has been described before [43]. The rabbit polyclonal antibody against HBc was purchased from Dako.

### Transient transfection

HepG2 cells in 60-mm dishes were transfected with 4 μg (total) of plasmid using X-tremeGENE HP DNA Transfection Reagent (Roche). HEK293T cells seeded in 60-mm dishes were transfected with 10 μg (total) of plasmid DNA using the CalPhos Mammalian Transfection Kit (Clontech). Cells and culture supernatant were harvested on day five post-transfection. Huh 7 cells in 100-mm dishes were transfected with 12 ug of (total) of plasmid DNA using the FuGENE 6 transfection reagent (Promega).

### HBV infection

HBV infection in HepG2-NTCP was carried out as previously described [29], with slight modifications. Briefly, HepG2-NTCP cells were plated in collagen I-coated 35-mm tissue culture dishes. When the cells reached 50% confluence, they were pretreated with 2% DMSO for 7 days to arrest cell growth. The cells were then infected by using the HBV inoculum harvested from induced HepAD38 cells or transiently transfected Huh7 cells, at a multiplicity of infection (MOI) of ca. 200–400 genome equivalent (GE)/cell in the presence of 2% dimethyl sulfoxide (DMSO), 4% polyethylene glycol (PEG) 8000, in the presence or absence of the indicated compounds. Freshly plated primary human hepatocytes isolated from chimeric mice containing human hepatocytes (PXB cell; PhoenixBio) were also used for HBV infection. PXB cells were plated on type I collagen-coated plates by PhoenixBio and cultured in modified dHCGM (DMEM with 10% FBS, 100 U/mL Penicillin, 100 ug/mL Streptomycin, 20 mM HEPES, 44 mM NaHCO_3_, 15 ug/mL L-proline, 0.25 ug/mL Insulin, 50 nM dexamethasone, 5 ng/mL EGF, 0.1 mM Asc-2P, 2% DMSO) [54]. Upon delivery, the cultured medium was replaced with fresh modified dHCGM. The cells were infected the next day (day 0) by replacing the culture medium with infection medium (dHCGM with 4% PEG 8000 and 5 ul inoculum harvested from HepAD38 cells at an MOI of 400 GE/cell. The inoculum was incubated with the cells for 20–28 hrs. Subsequently, the culture medium was changed daily until day 3 when they were harvested for analysis of HBV CCC DNA. To inhibit the CDK2 activity in PXB cells during HBV infection, the CDKII inhibitor K03861 (Selleckchem, cat no. S8100) or CDK2 inhibitor III (Sigma, cat no. 238803) [45] was added along with the HBV inoculum. The cells were harvested three days post infection.

### Induction of rapid and synchronous formation of HBV CCC DNA in HepAD38 cells

To test the role of CDK2 in intracellular CCC DNA amplification, we adopted the synchronized CCC DNA formation system using induced HepAD38 cells as reported recently [55]. Briefly, Tet was removed from the culture medium to induce transcription of HBV pgRNA. Two days after Tet removal, the reversible inhibitor of the HBV reverse transcriptase, phosphonoformic acid (PFA) [56, 57], was added to the culture medium at 2 mM concentration to allow synthesis of HBV SS DNA but not RC DNA. PFA treatment was maintained for four days to accumulate nucleocapsids containing SS DNA. PFA was then removed to allow synchronous synthesis of RC DNA and the resulting conversion of RC to CCC DNA, and Tet was added back simultaneously to block further pgRNA transcription. At the same time of PFA removal and Tet add back, the CDK2 inhibitor was added. Cells were harvested 24 hrs after PFA removal for analysis of HBV DNA.

### Isolation of viral DNA

The HBV core and PF DNAs were isolated from transiently transfected cells as previously described [28, 29] with minor modifications. For isolation of core DNA, cells were lysed in NP-40 lysis buffer (50 mM Tris-HCl [pH 8.0], 1 mM EDTA, 1% NP-40, and 1X protease inhibitor [Roche]). After removal of the nuclear pellet by centrifugation, the supernatant (cytoplasmic lysate) was incubated with micrococcal nuclease (MNase) (Roche) (150 units/ml) and CaCl_2_ (5 mM) at 37°C for 90 min to degrade the nucleic acids outside NCs. The MNase was then inactivated by addition of 10 mM EDTA. Thereafter, proteinase K (0.6 mg/ml) and sodium dodecyl sulfate (SDS) (0.5%) were used to digest and disrupt NCs and viral DNA-protein complexes. HBV DNA released from NCs was then purified by phenol-chloroform extraction and ethanol precipitation. Hirt extraction was used for PF DNA isolation. Cells in a 60-mm dish were lysed in 1 ml SDS lysis buffer (50 mM Tris-HCl [pH 8.0], 10 mM EDTA, 150 mM NaCl, and 1% SDS). After incubation for 5 min at room temperature, the cell lysate was transferred to a 1.5-ml microcentrifuge tube, mixed with 0.25 ml of 2.5 M KCl, and incubated at 4°C overnight with gentle rotation. After being centrifugated at 14,000 x g for 20 min, the supernatant was extracted three times with phenol and once with chloroform. The DNA was precipitated with ethanol and washed with 70% ethanol three times, vacuum dried, resuspended in 200 ul TE (10 mM Tris-HCl–1 mM EDTA [pH 8.0]).

### Exonuclease treatment

To remove the input plasmids from purified viral DNA, Dpn I digestion was used [10, 29]. The Dpn I-treated DNA was further digested with Exonuclease I (Exo I) and Exonuclease III (Exo III) (NEB) as described [29]. Briefly, 20 ul PF DNA (from total 200 ul per 6-cm dish of cells) was digested with 1 ul of Exo I (20 units) & 0.25 ul of Exo III (25 units) in the 1x Cutsmart buffer (NEB) at 37°C for 2–3 hrs [29].

### Southern blot analysis of viral DNA

The HBV core and PF DNAs were resolved on a 1.2% agarose gel and detected by Southern blot analysis as previously described [29, 53, 57], using a ^32^P-labeled HBV DNA probe or strand-specific RNA probes as indicated. Where indicated, viral DNA was also released from NCs in cytoplasmic lysate, without prior nuclease digestion or further purification, by SDS (0.6%)-proteinase K (0.5 ug/ul) digestion as described [26, 27]. The digested lysate was directly resolved by agarose gel electrophoresis and analyzed by Southern blot analysis.

### Capsid assembly and RNA packaging

To determine the levels of assembled capsids and the amount of pgRNA packaged inside capsids, cytoplasmic lysates were resolved on a 1% native agarose gel (native agarose gel electrophoresis or NAGE). Upon transfer of the resolved capsids onto nitrocellulose membrane, the packaged pgRNA was detected using a ^32^P-labeled anti-sense RNA probe as described previously [53]. Subsequently, capsids were detected on the same membrane by using the rabbit polyclonal (Dako) or mouse monoclonal (T2221) anti-HBc antibody as described [22].

### Virion secretion assay

To analyze HBV virion secretion, cell culture supernatant was first precipitated with polyethylene glycol (PEG) and subjected to DNase I digestion to remove plasmid DNAs [57, 58]. The concentrated viral particles were resolved on a 1% native agarose gel and detected using a ^32^P-labeled HBV DNA probe following transfer to nitrocellulose membrane. The same membrane was subsequently probed with the indicated core- or surface-specific antibody to detect the core or surface proteins, as described [22, 58].

### Endogenous kinase reaction (EKR)

EKR was performed as described before [45, 57] with minor modifications. Briefly, 20 ul NP-40 lysate (600 ul total per 60 mm dish) from transfected HEK293T cells was digested with proteinase K for 1 hr (final concentration, 1 mg/ml). Then, the proteinase K was stopped by adding the proteinase K inhibitor (Calbiochem) to 1 mM and incubating the sample at room temperature for 10 min. The digested lysate was then mixed with the kinase reaction buffer (final concentration: 10 mM sodium phosphate, pH 7.0, 10 mM MgCl_2_, and 0.01% Brij-35), 1x EDTA-free protease inhibitor cocktail (Roche), 1 mM dithiothreitol (DTT), and 5 uCi [γ-^32^P]ATP (3,000 Ci/mmol, 10 mCi/ml; Perkin Elmer) and incubated at 37°C for one hr. The reaction mixture was loaded on a 1% agarose gel. After transfer to nitrocellulose membrane, the radiolabeled capsids were detected by phosphorimaging and the total capsid levels determined by western blot analysis using the anti-HBc antibody T2221. In addition, the EKR reactions were also resolved by SDS-PAGE. After transfer to polyvinylidene difluoride membrane, radiolabeled HBc proteins were detected by phosphorimaging and total HBc proteins detected by the anti-HBc antibody T2221.

### Phos-tag gel electrophoresis

Phos-tag gel electrophoresis to separate different phosphor-isomer of HBc was conducted as described [50, 59, 60], with minor modifications. Briefly, cytoplasmic lysate containing HBc proteins was boiled in SDS sample buffer containing 1 mM MnCl_2_ and 10% β-ME for 10 min and then resolved on an SDS-polyacrylamide gel (10%, acrylamide:bis-acrylamide ratio 37.5:1) containing 100 uM Phos-tag acrylamide and 200 uM MnCl_2_. After electrophoresis, the gel was soaked in transfer buffer containing 10 mM EDTA with gentle shaking for 10 min each time, with one to three buffer changes, before western blot analysis.

### Quantification and statistical analysis

All experiments were repeated at least three times (three biological repeats, e.g., three separate transfections). Radioactive signals from Southern blot analysis and EKR were detected by phosphor imaging (Typhoon 9500, GE Healthcare Life Sciences) and quantified using Quantity ONE (Bio-Rad). Protein signals from western blot analysis were detected and quantified using the Bio-Rad Chemi-Doc (ImageLab) system. Subtraction of background signals was done via either the global method when the background signal was fairly uniform and was subtracted from all sample lanes, or done separately for each individual lanes using appropriate background within that particular lane. All quantifications were performed within the linear range of the instruments in the absence of signal saturation. To control for possible variations in transfection efficiency and sample loading, viral parameters from each batch of transfected cells at each step of viral replication were normalized to those from the preceding step from the same batch of transfected cells, e.g., capsid to total HBc levels for capsid assembly efficiency, pgRNA packaging to capsid levels for RNA packaging efficiency, core DNA to RNA packaging levels for DNA synthesis efficiency, CCC DNA to core RC DNA levels for CCC DNA formation efficiency, etc. The PEG precipitation procedure to concentrate secreted virions (as well as naked capsids) from the cell culture supernatant was found to generate consistent results from multiple experimental repeats and consistent precipitation efficiency from multiple samples could also be verified by staining of total proteins precipitated following resolution by SDS-PAGE.

## Results

### Rationale

**Fig 1:**
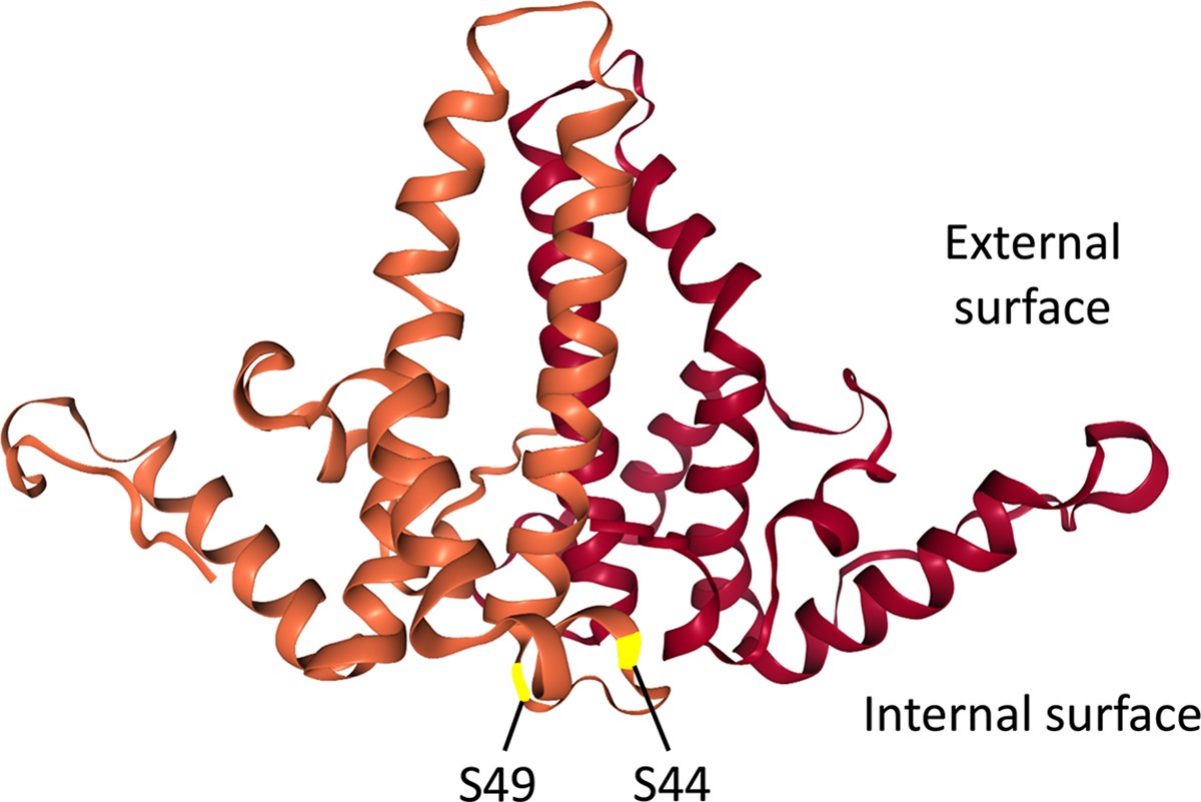
HBc dimer structure and location of the putative NTD phosphorylation sites S44 and S49. The structure of the two HBc NTD monomer (in brown and dark red, respectively) in an NTD dimer, based on the HBV capsid crystal structure [17], is shown. The two putative NTD phosphorylation sites S44 and S49, located on the interior surface of the capsid, are highlighted.

Close inspection of the HBc NTD sequences revealed two conserved potential phosphorylation sites, S44 and S49 (Fig 1), both in S-P motifs, which are localized to the interior surface of capsids. Two major classes of so-called proline-directed kinases, which are the major cellular protein kinases known to phosphorylate S(T)-P motifs, are CDKs and MAPKs [61]. Also, these two putative NTD phosphorylation sites would be inaccessible to cellular kinases once the capsid is assembled but would be accessible to a kinase that is packaged inside the capsid. Given our recent discovery that the major endogenous kinase packaged in HBV capsids is CDK2 (or at least a closely related kinase) [45], we hypothesized that the NTD SP motifs can be phosphorylated by the packaged CDK2 during certain stage(s) of viral replication to regulate HBc functions. To begin to test such a possibility, we changed both S44 and S49 to Ala (N2A) to block their phosphorylation and to Glu (N2E) to mimic constitutive phosphorylation (and block dephosphorylation). Using transient transfection assay in human hepatoma cells that support HBV replication, we analyzed the effects of these mutations on different stages of HBV replication and on the phosphorylation state of HBc.

### Both N2A and N2E mutants showed WT levels of capsid assembly but N2E showed decreased levels of RNA packaging

**Fig 2:**
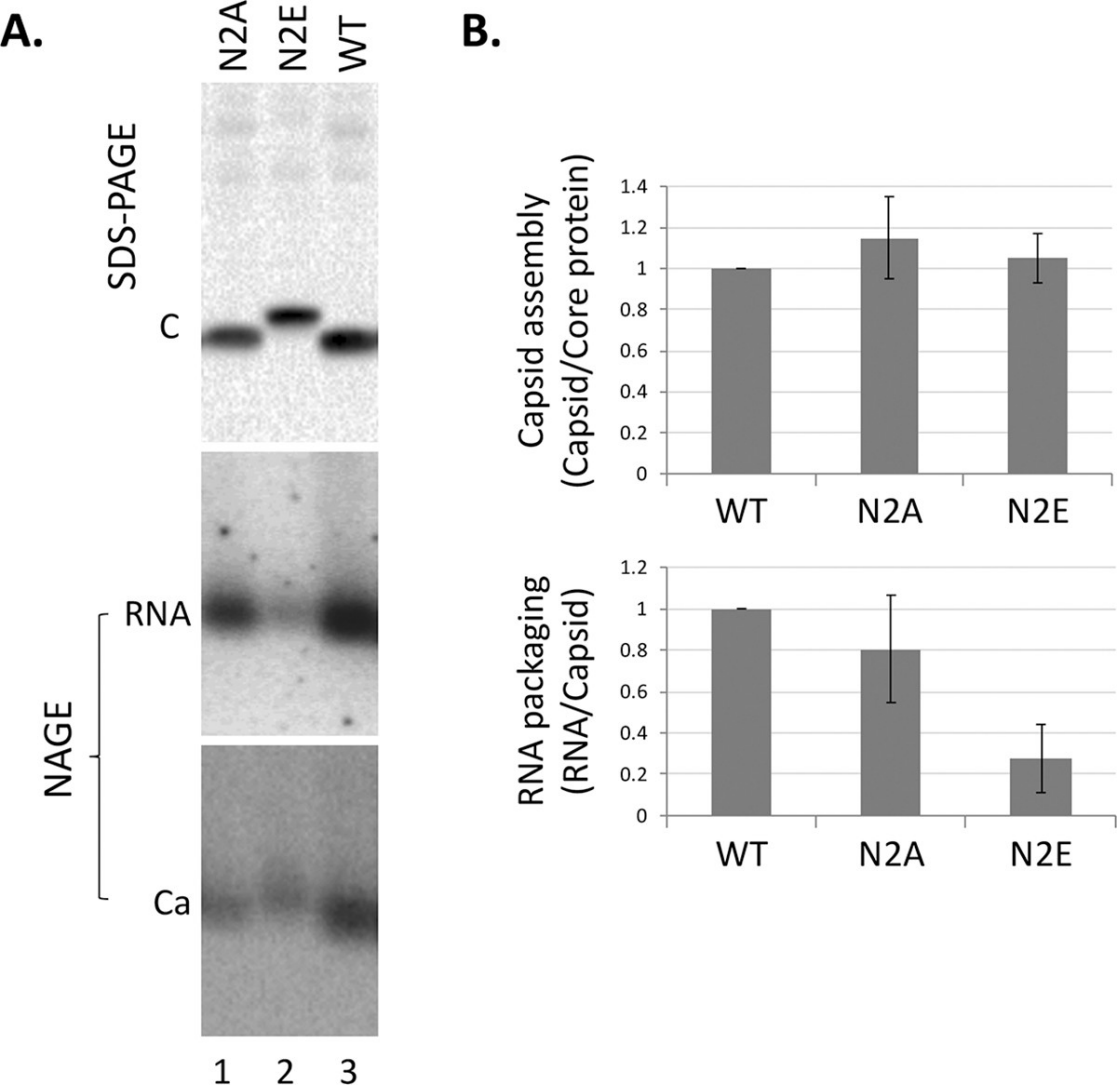
Effects of NTD phosphorylation mutants on capsid assembly and RNA packaging. The HBV replicon construct expressing the N2A or N2E mutant, or WT HBc was transfected into HepG2 cells. Transfected cells were lysed five days later. **A.** Levels of HBc proteins (top) were measured by western blot analysis using the T2221 anti-HBC NTD mAb following resolution by SDS-PAGE (top). Assembled capsids (bottom) and packaged RNA (middle) were detected by using a plus strand specific RNA probe and the Dako anti-HBc polyclonal antibody, respectively, following resolution by native agarose gel electrophoresis (NAGE) and transfer to nitrocellulose membrane. C, HBc protein; Ca, HBV capsid. RNA signals were detected by phosphorimaging scan and protein signals by chemiluminescence scan. **B.** Quantitative results from multiple experiments shown in **A**. Capsid assembly efficiency (top) was determined by normalizing the levels of capsids measured following NAGE to those of HBc proteins following SDS-PAGE, with the efficiency from WT HBc set to 1.0. RNA packaging efficiency (bottom) was determined by normalizing the levels of RNA packaging to those of capsids, with the efficiency from WT HBc set to 1.0.

Using an overlength HBV genomic construct (replicon, see Materials and Methods) that is competent to produce pgRNA and initiate viral replication in human hepatoma cells, we tested the effects of the NTD mutations on HBc expression, capsid assembly, pgRNA packaging, reverse transcription, CCC DNA formation, and virion secretion. SDS-PAGE and western blot analysis of lysate of transfected cells showed HBc expression levels were not affected by either the N2A or N2E mutation (Fig 2A, top). Interestingly, the N2E mutant protein migrated on SDS-PAGE significantly slower than the WT HBc, either due to enhanced phosphorylation or conformational change (see below).

Capsid assembly and pgRNA packaging were then determined using the NAGE assay. We have previously shown under our experimental conditions, mostly pgRNA, but not (+) strand DNA, was detected in our RNA packaging assay [53, 58]. To further verify that our RNA packaging assay was detecting mostly the packaged HBV RNA, but not DNA, we included analysis of capsids harvested from cells that were treated with the HBV RT inhibitor (entecavir, ETV), which is known to block viral DNA synthesis but not pgRNA packaging. While HBV DNA-associated with capsids, detected by a riboprobe specific for the minus strand DNA, was decreased dramatically in the entecavir treated samples (S1 Fig, lanes 7–9 vs 10–12), the packaged RNA levels, detected by a riboprobe specific for the HBV RNA (and plus strand DNA), were not significantly changed (S1 Fig, lanes 1–3 vs. 4–6). Both in the presence and absence of entecavir, RNA packaging by N2E was decreased by 4–5 fold compared to the WT HBc whereas N2A showed little effect (Fig 2A, middle and bottom; Fig 2B; S1 Fig). On the other hand, capsids levels were not significantly affected by either mutant. These suggested that the N2E, but not N2A, capsids were partially defective in pgRNA packaging.

### N2A and N2E showed no specific defect in reverse transcription or secretion of complete virions

**Fig 3:**
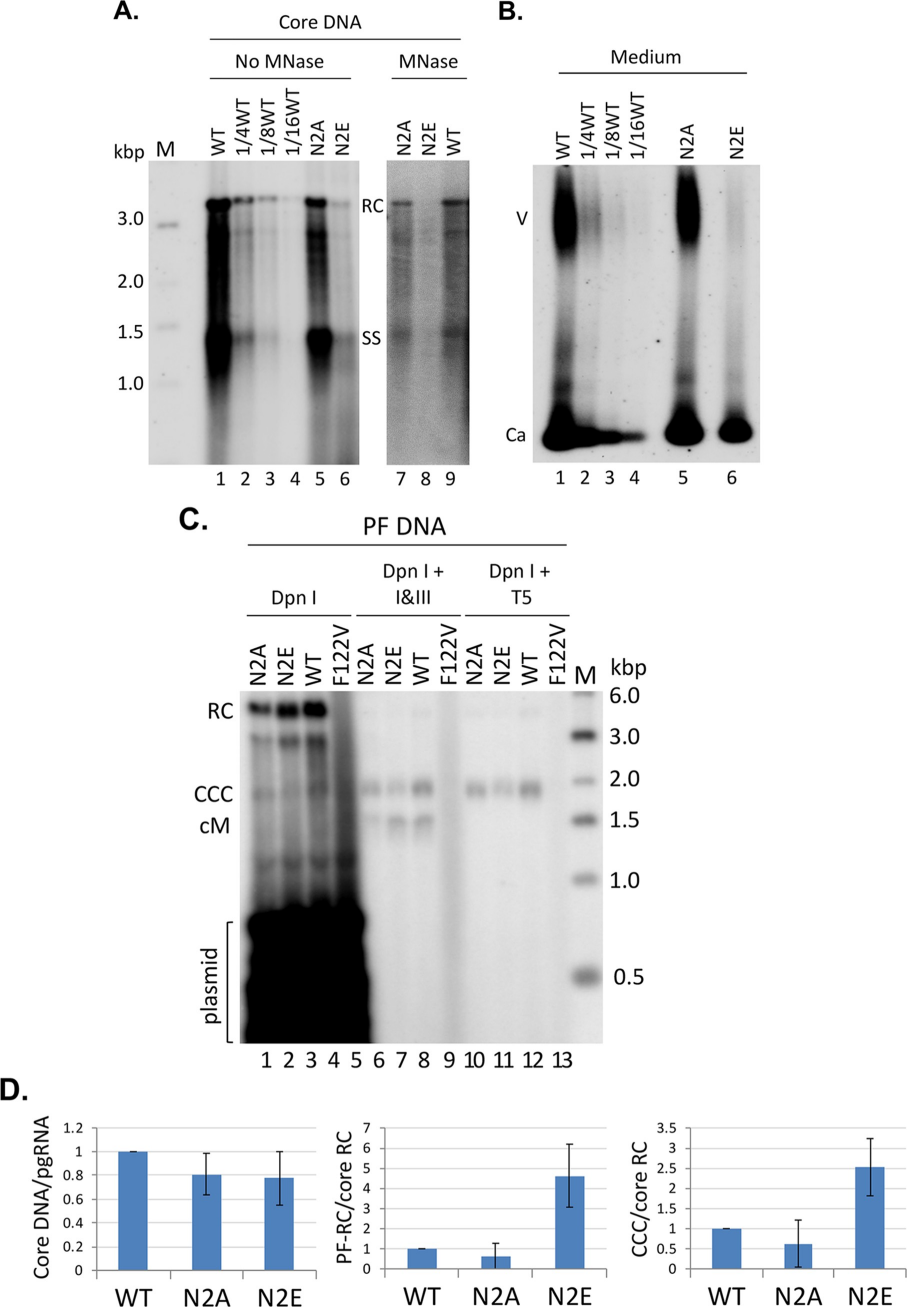
Effects of NTD phosphorylation mutants on core DNA and CCC DNA levels. HepG2 cells were transfected as in Fig 2. **A.** Cytoplasmic lysate from the transfected cells was treated with SDS-proteinase K to release the HBV core DNA from NCs, which was then resolved on an agarose gel (1% agarose) (lanes 1–6). In addition, a portion of cytoplasmic lysate was digested first with MNase to remove input plasmid DNA (and any core DNA not protected by the capsid) before SDS-proteinase K treatment. The core DNA was then purified and resolved on an agarose gel (lanes 7–9). Core DNA was then detected by Southern blot analysis using an HBV DNA probe. M, DNA size marker in kilo-basepairs (kbp); PI, plasmid DNA; RC, RC DNA; SS, single-stranded DNA. **B.** Viral particles released into the culture supernatant of the transfected HepG2 cells were concentrated by PEG precipitation and resolved on an agarose gel (1% agarose). Following transfer to nitrocellulose membrane, HBV DNA associated with virions (V) or naked capsids (Ca) was detected by Southern blot analysis using an HBV DNA probe. To facilitate a more clear visualization of the degree of N2E deficiency in DNA synthesis and virion secretion, as compared to the WT, serial dilutions (1/4^th^, 1/8^th^, 1/16^th^) of the WT samples were loaded (**A** and **B**, lanes 2–4). **C.** HBV PF DNA was extracted from the transfected HepG2 cells. The HBc F122V mutant (lane 4) defective in DNA synthesis was included as a negative control for PF DNA analysis. The extracted DNA was digested with Dpn I (to degrade the input plasmid DNA) (lanes 1–4), Dpn I plus the exonuclease I and III (I&III) (lanes 5–8), or Dpn I plus the exonuclease T5 (T5) (lanes 9–12) before resolution on an agarose gel (1.2% agarose) and detection by Southern blot analysis using an HBV DNA probe. M, DNA size marker in kilo-basepairs (kbp); RC, RC DNA; CCC, CCC DNA; cM, closed minus strand DNA. All Southern blot images shown in **A**, **B**, and **C** were from phosphorimaging scan. **D.** Quantitative results from multiple experiments. Left, levels of core DNA were normalized to those of RNA packaging measured in Fig 2; middle, PF-RC DNA normalized to core RC DNA; right, CCC DNA normalized to core RC DNA. All normalized DNA values from the WT HBc were set to 1.0.

To determine the nature and levels of the viral DNA synthesized from the packaged pgRNA in NCs, viral DNA was released from NCs (core DNA) by SDS/proteinase K treatment and detected by Southern blot analysis. Core DNA levels were obviously lower (by ca. 8-fold) in N2E NCs compared to the WT (Fig 3A, lanes 6 vs. 1–4), but this decrease could be largely accounted for by the decrease in pgRNA packaging shown in Fig 2 above. Upon normalization of core DNA levels to those of packaged RNA, neither N2E or N2A showed any significant reduction in core DNA (Fig 3D), thus indicating that these mutants showed little specific defect in reverse transcription.

HBV DNA levels in virions released into the culture supernatant were then analyzed by the NAGE assay. The amount of virion DNA detected by Southern blot analysis in the WT or the NTD mutants was proportional to their respective intracellular RC DNA levels (Fig 3A and 3B), indicating no specific block in the secretion of complete virions by the mutants. Specifically, the N2E virion DNA levels were reduced by ca. 8-fold compared to the WT, similar to the reduction in N2E intracellular DNA levels. Similarly, the amount of HBV DNA in naked capsids, which are also released into hepatoma cell culture supernatant, was also proportional to the intracellular HBV DNA levels.

### N2E but not N2A increased PF-RC and CCC DNA levels during intracellular amplification

The potential effects of the HBc NTD mutations on HBV CCC DNA formation was then analyzed. PF-DNAs, including PF-RC DNA and CCC DNA, extracted from transfected cells were analyzed by Southern blot analysis. Transfection input plasmid DNAs were digested by Dpn I. To further analyze the nature of PF DNA, Exo I/III or T5 nuclease digestion was carried out, which removes RC DNA [29]. T5 will also removes any RC DNA in which only one of the two strands is covalently closed due to its SS DNA endonuclease activity. As a negative control to monitor the digestion of non-viral DNA, another HBc mutant, F122V, which is defective in pgRNA packaging and viral DNA synthesis [62], was included in the analysis. HBV PF DNA levels were determined by Southern blot analysis and normalized to those of the core RC DNA, which is the ultimate precursor to both PF-RC DNA and CCC DNA. The results showed that levels of PF-RC DNA from N2E was increased by ca. 5-folds compared to the WT. Furthermore, CCC DNA levels from N2E were also increased by ca. 2.5-folds (Fig 3C and 3D). These results indicated that N2E produced PF-RC DNA and CCC DNA more efficiently. On the other hand, N2A tended to have less PF-RC and CCC DNA than WT although the difference did not reach statistical significance; the PF DNA levels from N2A appeared to be rather variable from experiment to experiment (Fig 3D) (see also Discussion below). We noticed a DNA species migrating at ca. 3.0 kb in the core DNA and PF DNA (Fig 3A and 3C; also see below). This could represent the minor form of the HBV genome, the double-stranded linear DNA as suggested earlier (10) but could also be an incomplete RC DNA species with a relatively short plus strand.

### The N2A mutant virus was impaired in CCC DNA formation during de novo infection

**Fig 4:**
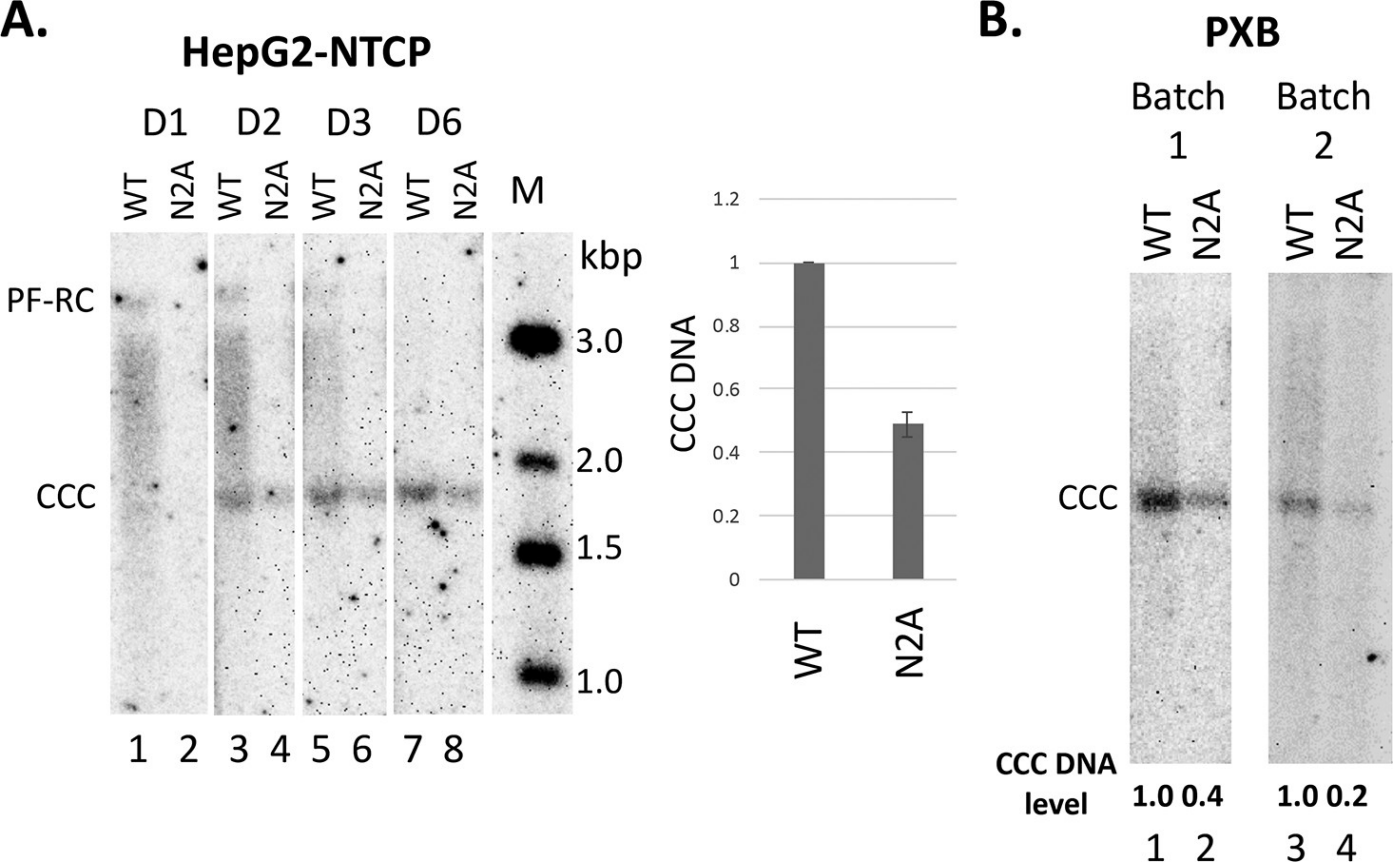
Effects of N2A on CCC DNA formation during infection. The WT or N2A mutant virus inoculum was prepared from transiently transfected Huh7 cells. The WT and N2A replicon constructs were transfected into Huh7 cells. The culture supernatant was harvested on day 5, 7 and 9 post-transfection, pooled and concentrated by PEG precipitation as described in the Methods. HepG2-NTCP and PXB cells were infected with the WT or N2A mutant virus. HBV PF DNA was extracted from the infected cells and measured by Southern blot analysis using a ^32^P-labeled HBV DNA probe. The Southern blot images shown were from phosphorimaging scan. **A.** A representative Southern blot autoradiogram of PF DNA from HepG2-NTCP cells extracted at the indicated days post-infection. Quantitative analysis of CCC DNA levels at day three post-infection from multiple infection experiments is presented in the graph to the right, with the CCC DNA level from WT HBV infected cells set to 1.0. M, DNA m.w. markers in kbp; **B.** Representative Southern blot autoradiograms of PF DNA from two batches of PXB cells extracted three days post-infection. Relative CCC DNA levels are indicated at the bottom of the autoradiograms with the CCC DNA level from WT HBV infected cells set to 1.0.

To test if the NTD state of phosphorylation played a role in CCC DNA formation during viral infection, we prepared the WT and N2A mutant virus inoculum using transient transfection of Huh7 cells. Unfortunately, the N2E mutant secreted too little virus and it was thus difficult to make sufficient amounts of the N2E mutant virus for the infection experiments. We then infected both the HepG2-NTCP cells and PXB cells (primary human hepatocytes isolated from human liver-chimeric mice) [54] with WT or N2A mutant virus at the same MOI. As described in the Methods, HepG2-NTCP cells were pretreated with DMSO to arrest their growth so as to mimic the non-dividing hepatocytes in the human liver before infection. Analysis of HBV CCC DNA levels three days post-infection showed that the N2A mutant virus was clearly less efficient than the WT in CCC DNA formation during infection of the HepG2-NTCP cells (Fig 4A) and both batches of PXB cells (Fig 4B).

### Mature NCs of the N2E mutant was destabilized compared to WT

The decrease in core DNA accompanied by the increase in PF-RC DNA and CCC DNA shown by N2E was reminiscent of other HBC NTD mutants that we reported before (such as L60A and I126A) [26], which cause hyper-destabilization (loss of integrity) of mature NCs (and thus more efficient uncoating than the WT) and more efficient release of mature RC DNA into the host cell for conversion to PF-RC DNA and CCC DNA. To test if N2E or N2A affected the integrity of NCs, we tested the ability of the mutant NCs to protect their DNA content from exogenous nuclease digestion as we described before [26, 27, 63]. Indeed, the N2E mutant failed to protect its RC DNA while able to protect the immature DNA species (Fig 3A, lane 8 vs. 6), indicative of a selective destabilization of mature NCs. In contrast, N2A protected both the RC and immature DNAs just like the WT HBc (Fig 3A, lanes 7 and 9 vs. 5 and 1).

### The N2E mutant increased the production of cM-RC DNA more than CCC DNA

**Fig 5:**
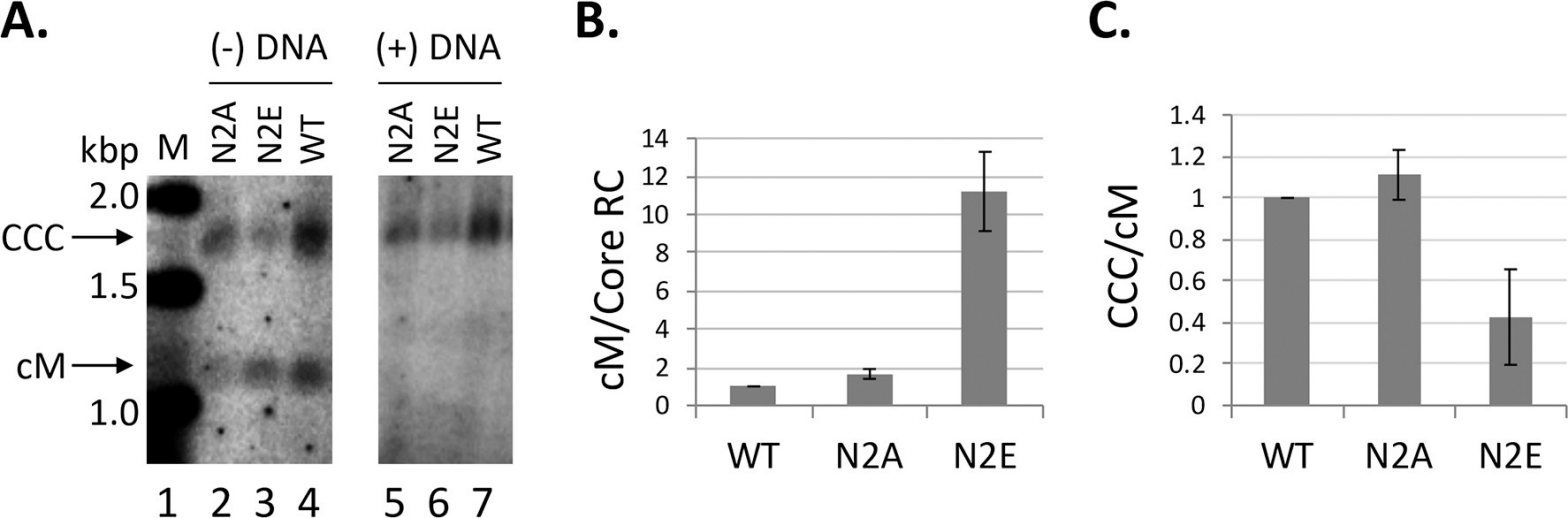
NTD phosphorylation could affect cM-RC DNA formation. The same PF-DNA samples shown in Fig 3. were treated with the exonuclease I and III before detection by Southern blot analysis, using strand specific riboprobes to detect either the minus (-) or plus (+) strand DNA separately (**A**). M, DNA size marker in kilo-basepairs (kbp); CCC, CCC DNA; cM, closed minus strand DNA. The Southern blot images shown were from phosphorimaging scan. **B** and **C.** Quantitative results from multiple experiments. **B**. cM-RC DNA normalized to core RC DNA. **C.** CCC DNA normalized to cM-RC DNA. All normalized values from the WT HBc were set to 1.0.

As we reported recently [29], a comparison of Exo I plus III vs. T5 nuclease digestion revealed the existence of the cM-RC DNA, as evidenced by the detection of a minus strand circular DNA (cM) upon digestion of the PF-RC DNA, which was preserved following Exo I plus III treatment but removed by Exo T5 (Fig 3C). Interestingly, the ratio of cM DNA to CCC DNA appeared to be affected by the NTD mutations. In particular, the N2E mutant produced more cM DNA relative to CCC DNA, as compared to the WT whereas N2A produced similar amounts of cM DNA to WT (Fig 3C, lanes 5–7). To verify that these circular SS DNA bands were indeed derived from minus strands but not plus strands (which could arise due to nicking of CCC DNA), we performed the Southern blot analysis of the PF DNA using the minus strand- or plus strand-specific RNA probe. The results showed that the circular SS DNA was only of minus (but not plus) strand polarity (Fig 5A), indicating it was indeed derived from the cM-RC DNA following removal of the open plus strands [29]. After normalization of the cM-RC DNA to core RC DNA (the precursor to cM-RC DNA), N2E showed ca. 10-fold increased levels of cM-RC DNA as compared to WT (Fig 5B). Also, after normalization of levels of CCC DNA (which is likely the product from cM-RC DNA) to those of cM-RC DNA, N2E showed ca. 2.5-fold decreased cM-RC DNA compared to the WT (Fig 5C). These results thus suggested that N2E preferentially stimulated the production of cM-RC DNA, an early step in CCC DNA formation, but didn’t simulate as strongly as (or might even have inhibited) the later step of cM-RC DNA to CCC DNA conversion. Again, the N2A mutant didn’t significantly affect the levels of CCC DNA or cM-RC DNA via intracellular amplification. These results suggested that the core protein may modulate different steps in CCC DNA formation prior to the covalent closing of the minus strand as well as subsequent steps following cM-RC DNA formation (see Discussion below).

### N2E capsids showed increased levels of phosphorylation in vitro by the endogenous kinase

**Fig 6:**
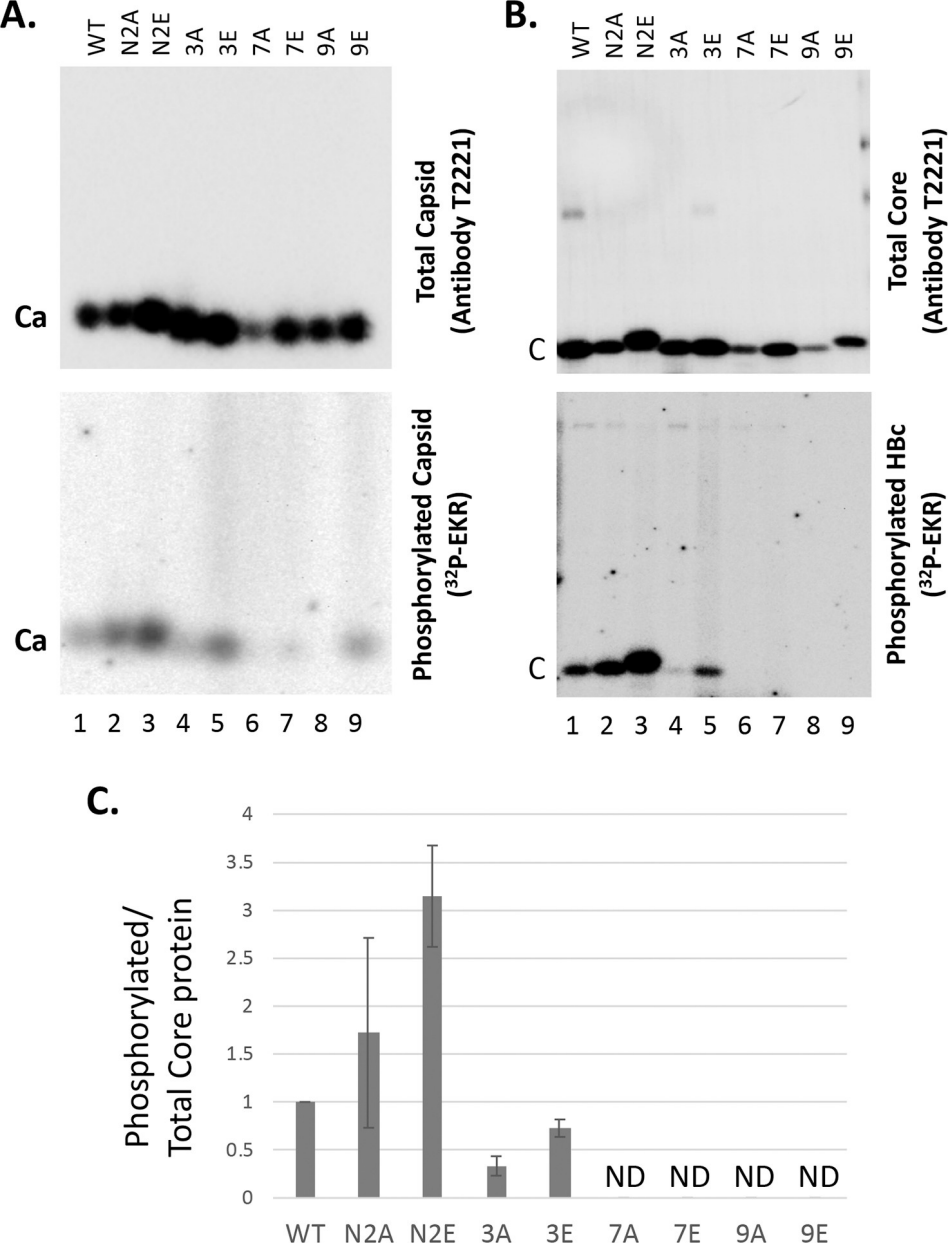
EKR using WT and mutant HBV capsids. The WT and mutant HBc expression constructs were transfected into HEK293T cells. In addition to the N2A and N2E mutants above, additional HBc mutants tested here included 3A/3E (with three major CTD sites of phosphorylation—all SP motifs—changed to A or E) [30], 7A/7E (with all seven CTD sites of phosphorylation changed to A or E), and 9A/9E (with all seven CTD sites as well as the two putative NTD-S44/S49 sites changed to A or E). Cytoplasmic lysate was prepared from the transfected cells using 1% NP-40 five days after transfection. The lysate was treated with 0.5 ug/ul proteinase K at 37°C for one hr before EKR in the presence of [γ-^32^P]ATP. The reaction products were resolved on the native agarose gel (**A**) or by SDS-PAGE (**B**). Total capsid (**A**) or total core protein (**B**) levels were measured by chemiluminescence western blot assay using the HBc antibody T2221 (top). Radiolabeled (phosphorylated) capsid or core protein levels resulting from the EKR were measured using phosphorimaging (bottom). Ca, capsids; C, HBc protein (subunit). **C**. Phosphorylation efficiency during EKR was determined by normalizing the levels of labeled core protein to total core protein from **B**, with that from the WT capsid set to 1.0. ND, not detectable.

To test whether the HBc NTD S44/S49 sites could serve as phosphorylation sites by the endogenous kinase in HBV capsids, we isolated WT and mutant HBc capsids from human HEK293T cells and conducted the EKR in vitro. In addition to N2A and N2E, we also isolated several additional mutant capsids, including 3A/3E (with three major CTD sites of phosphorylation changed to A or E) [30], 7A/7E (with all seven CTD sites of phosphorylation changed to A or E) [30], and 9A/9E (with all seven CTD sites as well as the two putative NTD-S44/S49 sites changed to A or E), for the EKR analysis. It was notable that the EKR signal from N2E was stronger than WT (Fig 6), suggesting that phosphorylation of these two NTD sites might stimulate CTD phosphorylation. Indeed, all other E mutants tested (3E, 7E, 9E) showed higher EKR phosphorylation signals than the corresponding A mutants (Fig 6). These results suggested that HBc phosphorylation might be cooperative and coordinated (see also Discussion below). The 3E mutant showed EKR signals nearly as high as WT, suggesting that the four minor in vivo CTD sites of phosphorylation, as well as any other putative sites of phosphorylation outside CTD, were used in the EKR efficiently. That the 7A and 9A mutants showed almost no EKR signal and the 7E and 9E showed only very low EKR signals suggested that the endogenous kinase mostly phosphorylated CTD. Although the residual EKR signals from 7E and 9E on the agarose gel (Fig 6A) suggested that HBc could be phosphorylated by the endogenous kinase outside CTD during EKR, phosphorylation of the mutant HBc protein itself could not be confirmed by SDS-PAGE for these mutants (Fig 6B). Therefore, it remained possible that the EKR signals detected on the agarose gel (i.e., associated with the intact capsid) (Fig 6A) might be due to phosphorylation of some other capsid-associated protein(s), although no other significantly phosphorylated proteins were detected by SDS-PAGE in Fig 6B as we reported previously [45]. On the other hand, it was also possible that the 7E and 9E mutant HBc proteins were phosphorylated (i.e., outside CTD) during the EKR in vitro but only at very low levels, which were below the limit of detection by the SDS-PAGE analysis.

### Phos-tag gel system revealed a high and variable degree of phosphorylation of the WT and mutant HBc proteins

**Fig 7:**
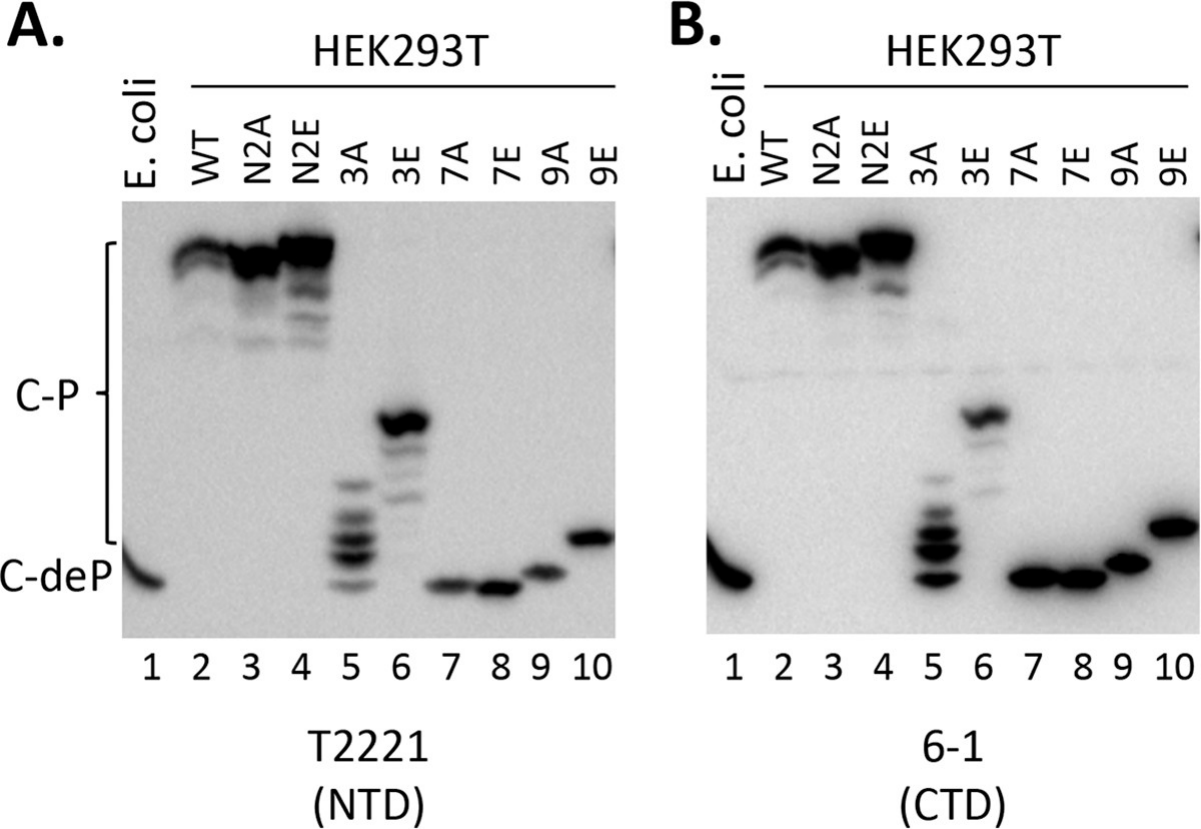
Phos-tag SDS-PAGE analysis of WT and mutant HBc proteins. HEK293T cells were transfected and cytoplasmic lysate from transfected cells were prepared as in Fig 5. The lysate was resolved on the Phos-tag gel, and HBc proteins were detected by chemiluminescence western blot assay using the mAb T2221 (HBc NTD) (**A**) or 6–1 (HBc CTD) (**B**). The HBc protein (non-phosphorylated) expressed and purified from E. coli was include as a control (lane 1). C-P, phosphorylated HBc; C-deP, dephosphorylated (non-phosphorylated) HBc.

The EKR results above suggested that the N2E mutant might increase HBc phosphorylation in cells. To look further into this possibility, we adopted the Phos-tag gel system to analyze the HBc phosphorylation level for the WT and mutant HBc expressed in human cells in the absence of viral replication, which can affect HBc state of phosphorylation (see Introduction). Protein mobility on this type of gel is directly proportional to the degree of phosphorylation [50, 59, 60]. Compared to the non-phosphorylated HBc expressed in E coli, the predominant protein species of WT, N2A, and N2E HBc proteins from human cells all migrated much slower (Fig 7, lanes 1–4), consistent with a high degree of phosphorylation of these proteins in human cells, as shown recently for the WT HBc [50]. A few minor, less phosphorylated species, which migrated faster than the predominant species from human cells but still much slower than HBc from E. coli, were also detectable for all three proteins. Furthermore, consistent with enhanced phosphorylation of N2E than WT or N2A, the major N2E species migrated even slower than the WT HBc and N2A migrated similarly to the WT, as observed above with regular SDS-PAGE analysis (Fig 2A).

We also included the HBc 3A, 3E. 7A, 7E, 9A and 9E mutants isolated from human cells in the Phos-tag gel analysis. In contrast to the relatively homogenous (and high) degree of phosphorylation of WT, N2A and N2E, 3E, and esp. 3A exhibited a much lower but highly heterogeneous pattern of phosphorylation, as evidenced by the multiple intense bands migrating well below the predominant WT (or N2A or N2E) band but still migrating slower than HBc from E. coli (Fig 7, lanes 5, 6). The much slower mobility of 3E compared to 3A on the Phos-tag gel again indicated the much stronger phosphorylation 3E compared to 3A in human cells, consistent with the in vitro EKR results above (Fig 6). 7A and 7E, with all CTD sites of phosphorylation eliminated, migrated like the non-phosphorylated HBc from E. coli, indicating the NTD sites S44/S49 (or any other potential phosphorylation sites in NTD or the linker of HBc) were not phosphorylated to any significant degree in human cells in the absence of NC maturation (Fig 7, lanes 7, 8). Interestingly, the 9E mutant migrated clearly slower than 7A/7E or the E. coli HBc (Fig 7, lane 10), suggesting that phosphorylation at the NTD S44/S49 sites could stimulate HBc phosphorylation outside CTD, i.e., other potential sites within NTD and/or the linker, consistent with the EKR results above (Fig 6). The reason for the slightly slower mobility of 9A (Fig 7, lane 9) relative to 7A/7E remains unclear but could suggest that there was residual HBc phosphorylation in 9A but not in 7A or 7E.

To exclude the possibility that the multiple HBc species detected on the Phos-tag gel were derived from degradation from the C-terminal end, which could conceivably occur due to the unstructured nature of the HBc CTD, we used two different anti-HBc antibodies to detect the HBc proteins resolved by the Phos-tag gel in Fig 7. In Fig 7A, the HBc proteins were detected using the NTD mAb, T2221, targeted to an epitope around HBc position 130–140 [22], which should detect all HBc proteins irrespective their state of phosphorylation. In Fig 7B, we used the mAb 6–1, which is directed at an epitope at the extreme C-terminal end of HBc (requiring the last HBc residue Cys183) [30]. The mAb 6–1 detected essentially all the HBc species detected by T2221, indicating that there was no significant C-terminal degradation and the multiple species detected following the Phos-tag gel resolution mostly, if not exclusively, represented the various HBc phosphor-isoforms (see Discussion below).

### Confirmation of enhanced CTD phosphorylation of N2E by phosphorylation state-specific antibodies

**Fig 8:**
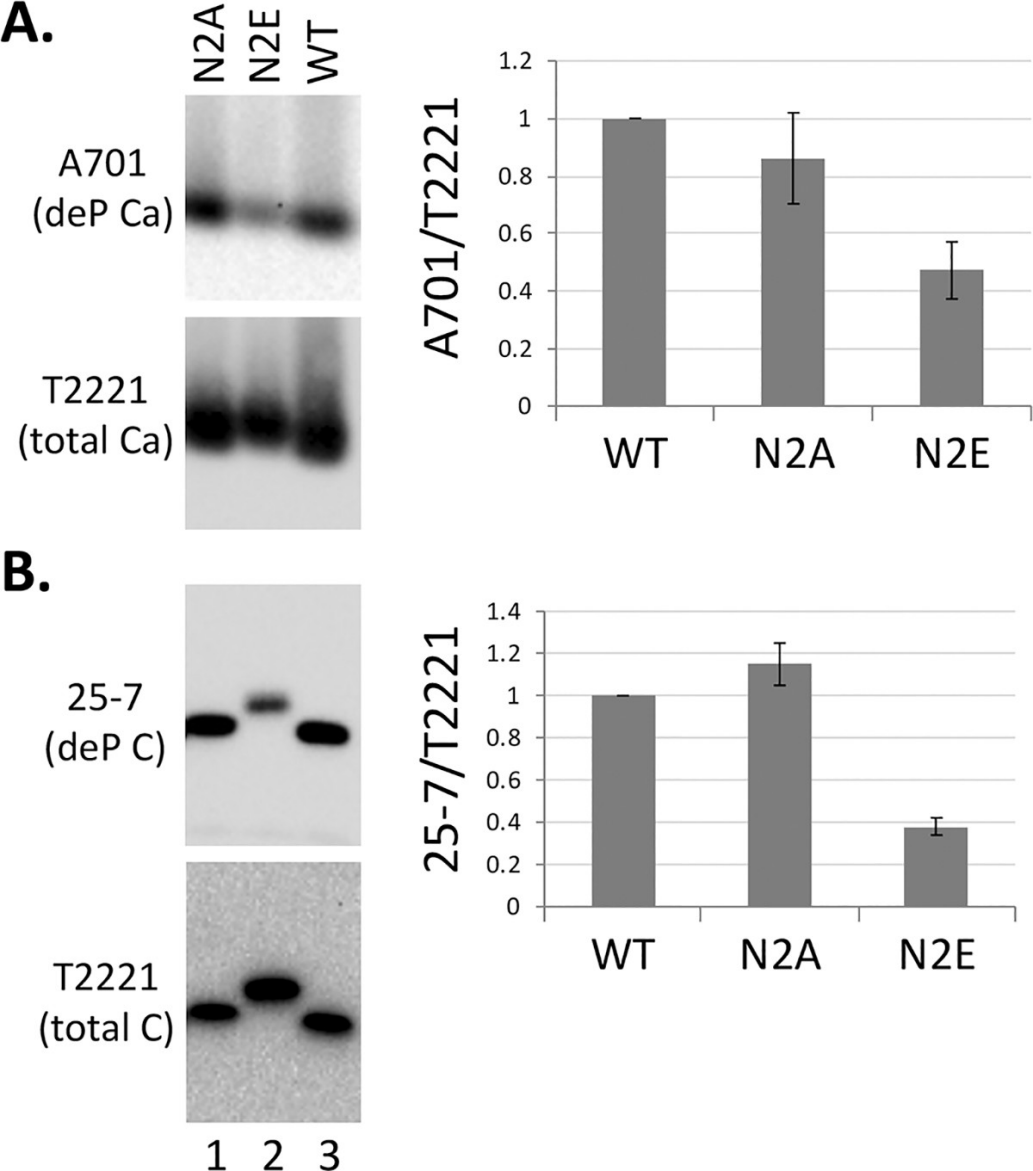
Analysis of effects of NTD mutations on CTD phosphorylation using CTD-phosphorylation state specific antibodies. HepG2 cells were transfected and cytoplasmic lysate from transfected cells were prepared as described in Fig 2. The lysate was resolved on a native agarose gel (**A**) or by SDS PAGE (**B**). HBc proteins were detected by chemiluminescence western blot analysis using the indicated mAbs selective for dephosphorylated (de-P) HBc (mAb A701 and 25–7) or with no selectivity for the phosphorylation state of HBc (total HBc) (mAb T2221). Quantitative results from multiple experiments are shown to the right. Levels of dephosphorylated HBc were normalized to those of total HBc, with the normalized value from the WT HBc set to 1.0. C, HBc protein; Ca, Capsid.

The in vitro EKR results as well as the Phos-tag gel analysis indicated that N2E, mimicking HBc NTD phosphorylation, could stimulate CTD phosphorylation by the endogenous kinase and in host cells. Taking advantage of our recently developed CTD specific mAbs that selectively recognize phosphorylated vs. non-phosphorylated CTD [22, 30, 43], we determined directly the state of CTD phosphorylation of WT, N2A, and N2E HBc proteins. Thus, WT and mutants HBc proteins expressed in human cells were resolved by SDS-PAGE (total HBc proteins, assembled and any free subunits) or agarose gel electrophoresis (only assembled capsids) and detected using different CTD antibodies as well as the NTD mAb T2221, which was used to detect all HBc proteins (irrespective phosphorylation state) for normalization of phosphorylated or non-phosphorylated HBc proteins. The mAbs selective for phosphorylated CTD mAbs didn't reveal significant differences among the different HBc proteins, likely due to the fact that the vast majority of the WT HBc protein expressed in human cells are normally heavily phosphorylated at CTD (Fig 7 above) [30, 43, 50, 60] such that any increase in CTD phosphorylation in N2E would not be dramatic (e.g., from 90% to 95% of the total protein). In contrast, a decrease in the (normally minor) non-phosphorylated CTD (from 10% to 5% in the above example) could be readily revealed, as we could demonstrate here (Fig 8). Thus, the mAb A701, selective for a non-phosphorylated CTD epitope from HBc 156–164 (including the major phosphorylation site S162 and the minor site T160), reacted less strongly with N2E capsids as compared to WT but with N2A capsids similarly to WT (Fig 8A), indicating that S162 (and possibly T160) was indeed more phosphorylated (less dephosphorylated) in N2E capsids than WT or N2A capsids. Similarly, mAb 25–7, which specifically recognizes a non-phosphorylated CTD epitope from HBc 165–183 (including the major phosphorylation site S170 and three minor sites S168, S176, and S178), reacted less strongly with the N2E protein compared to WT but with N2A similarly to WT (Fig 8B), indicating that one or more of phosphorylation sites within the 25–7 epitope were more phosphorylated in the N2E protein than WT or N2A. These results thus directly verified that N2E could stimulate CTD phosphorylation at least two different CTD sites.

### CDK2 inhibitors could block CCC DNA formation during both HBV infection and intracellular CCC DNA amplification

**Fig 9:**
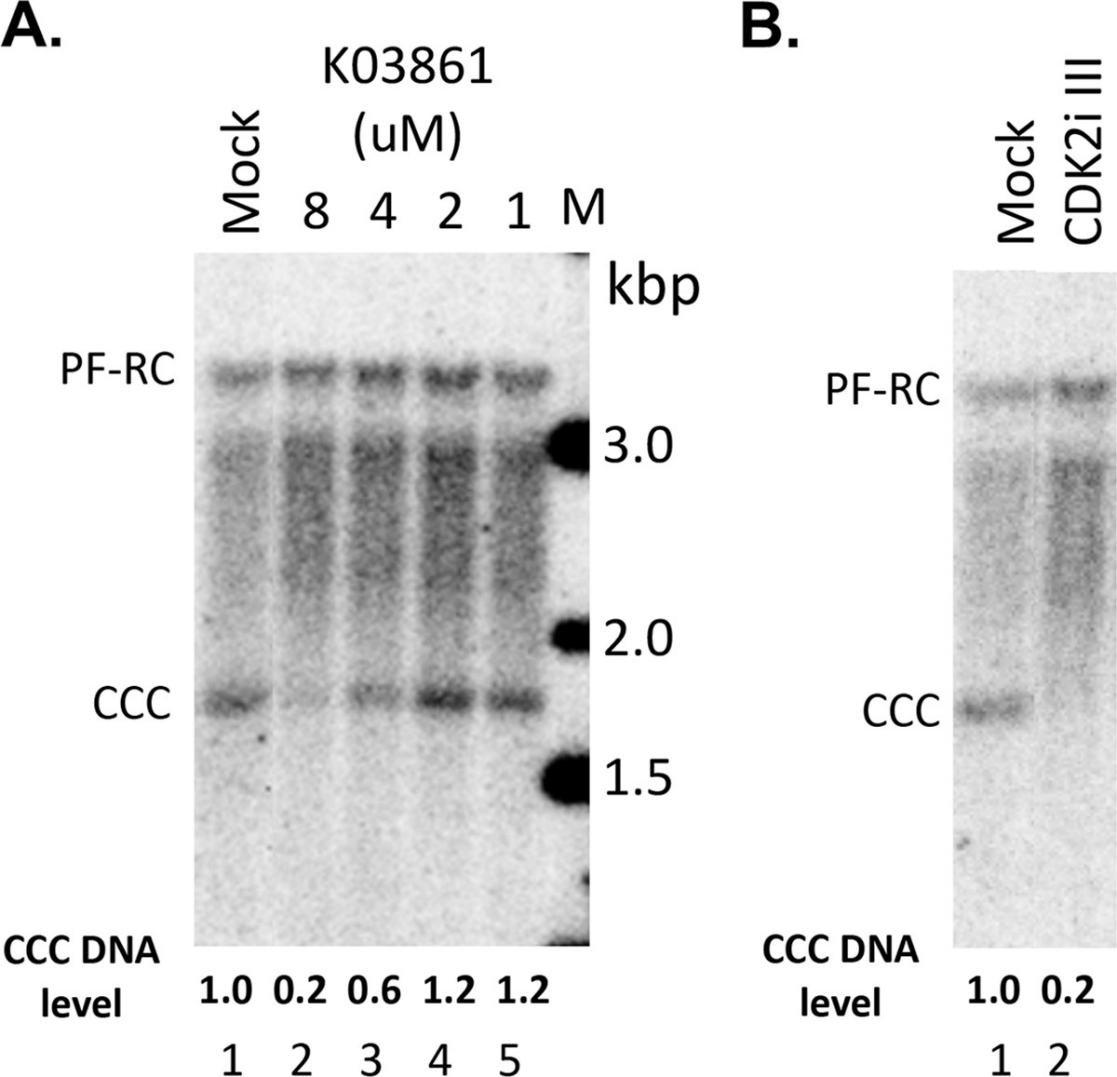
Effects of CDK2 inhibitors on CCC DNA formation during HBV infection. The PXB cells were infected with HBV and treated with the CDK2 inhibitor K03861 at the indicated concentrations (A) or CDK2 inhibitor III (CDK2i III; 125 nM) (B) at the same time. HBV PF DNA was extracted from the cells three days after infection and measured by Southern blot analysis using a ^32^P-labeled HBV DNA probe. Shown are representative Southern blot autoradiograms (phosphorimaging scan) of PF DNA, with the relative levels of CCC DNA indicated at the bottom and CCC DNA level from the mock-treated cells set to 1.0.

To test specifically the role of CDK2 in CCC DNA formation during HBV infection, via phosphorylation of mature NCs to stimulate uncoating as suggested above, we added two distinct CDK2 inhibitors, K03861 that acts by blocking cyclin binding to CDK2 [64], and the CDK2 inhibitor III that is an ATP-competitive inhibitor [45], during HBV infection of the PXB cells, primary human hepatocytes harvested from human liver-chimeric mice [54]. Both of these CDK2 inhibitors, as well as a broad-spectrum CDK inhibitor (roscovitine), could inhibit the endogenous kinase packaged inside the HBV capsid as determined by the EKR assay (S2 Fig), as we previously reported for the CDK inhibitor III and roscovitine [45]. Analysis of HBV CCC DNA levels in the infected cells three days post-infection showed that both CDK2 inhibitors could decrease CCC DNA formation during infection (Fig 9).

**Fig 10:**
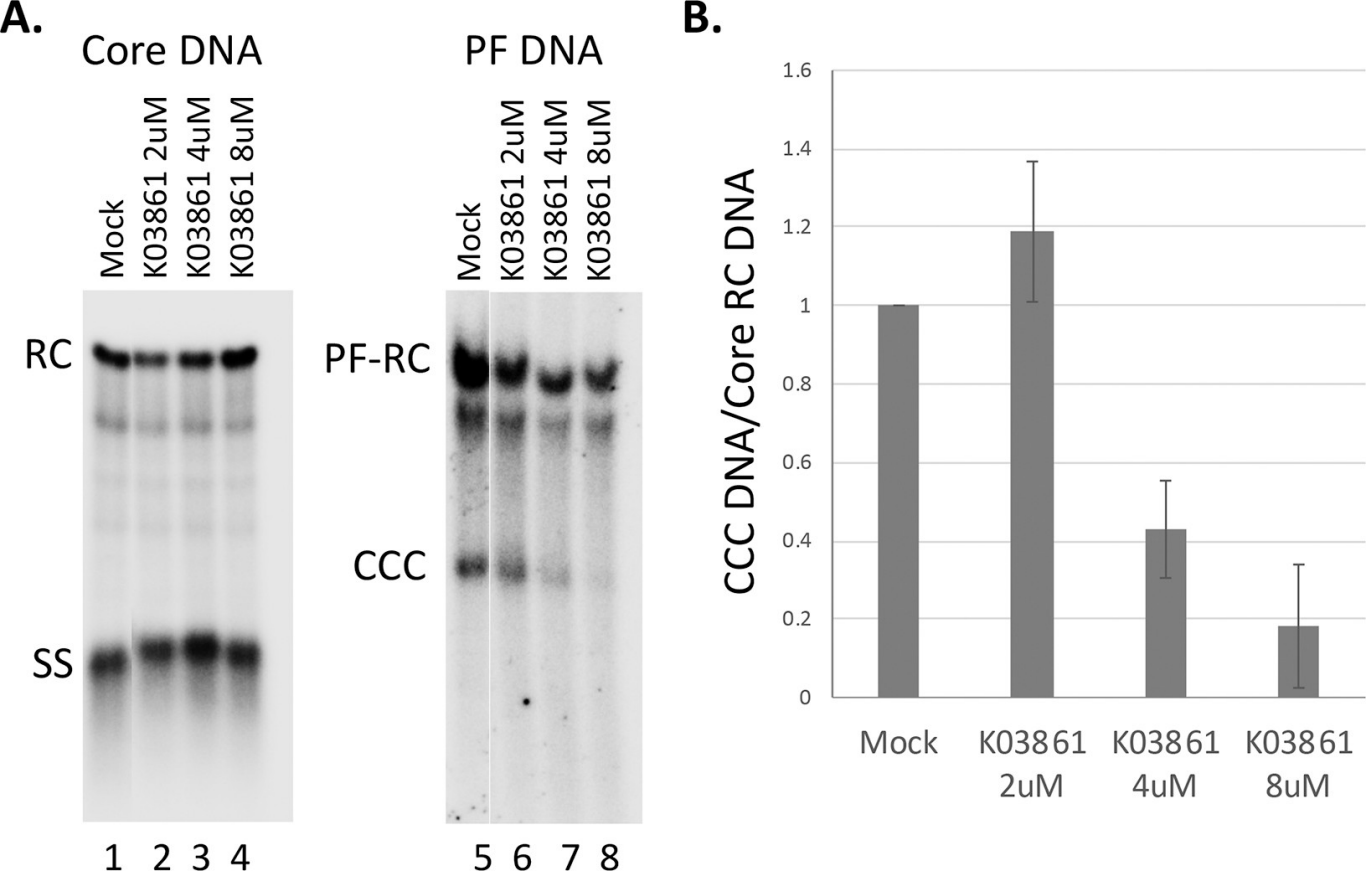
Effects of CDK2 inhibition on CCC DNA formation via intracellular amplification in HepAD38 cells. HBV pgRNA transcription were induced in HepAD38 cells by Tet removal. To accumulate SS HBV DNA, PFA was added into the culture medium on day two after Tet removal and maintained for the next four days. PFA was then removed to allow the synthesis of RC DNA and formation of CCC DNA. At the same time of PFA removal, the CDK2 inhibitor (K03861) was added. Twenty four hours later, HBV core DNA and PF DNA were extracted from the cells and measured by Southern blot analysis using a ^32^P-labeled HBV DNA probe. **A.** Representative Southern blot autoradiograms (phosphorimaging scan) of HBV core DNA (lanes 1–4) and PF DNA (lanes 5–8). **B.** Quantitative analysis of Southern blot results from multiple independent experiments. Data are expressed as CCC DNA levels normalized to those of core RC DNA, with the normalized CCC DNA level from the mock-treated cells set to 1.0.

To determine the role of CDK2 in CCC DNA formation during intracellular amplification, we also adopted the synchronized CCC DNA formation system using induced HepAD38 cells, as recently reported [55], which allows rapid HBV CCC DNA formation via intracellular amplification within one day (see Materials and Method for details). This rapid CCC DNA synthesis system helps to avoid potential cytotoxic or pleiotropic effects associated with prolonged CDK2 inhibition in cells. As shown in Fig 10, inhibition of CDK2 activity was able to suppress HBV CCC DNA formation, in a dose-dependent manner, during intracellular amplification, as during de novo HBV infection.

## Discussion

**Fig 11:**
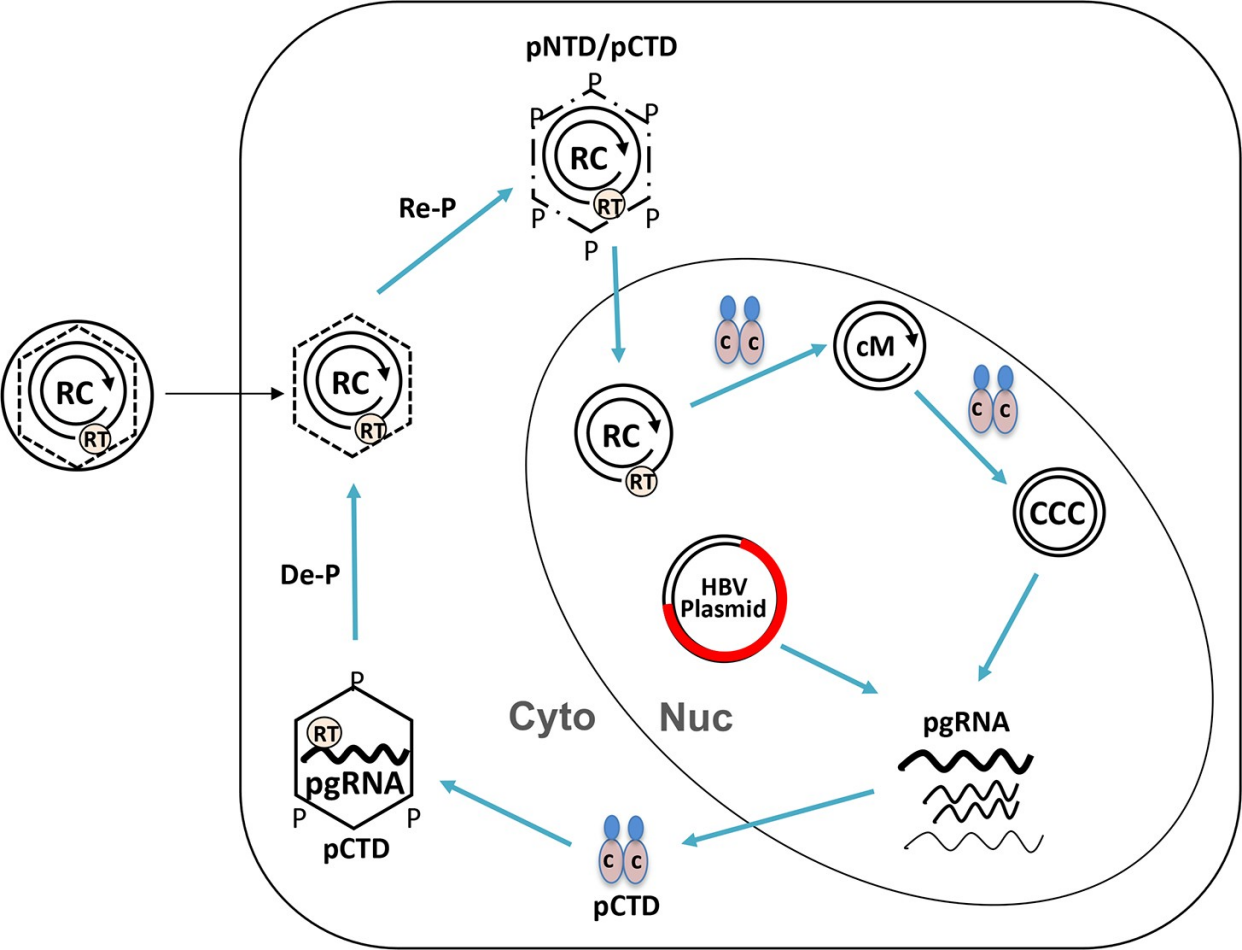
Model of HBc phosphorylation-dephosphorylation-rephosphorylation cycle in regulating NC assembly, maturation, and disassembly. Upon translation from pgRNA, HBc subunits are phosphorylated at CTD (pCTD) and assemble into immature NCs incorporating the RT protein and pgRNA in the cytoplasm (Cyto). During NC maturation, pgRNA is converted to RC DNA by the RT protein within maturing NCs while they undergo dephosphorylation to facilitate RC DNA synthesis and stabilization of mature NCs, although mature NCs are relatively unstable as compared to immature NCs (dashed vs. solid line of the hexagon). Rephosphorylation of HBc, at both NTD and CTD (pNTD/pCTD), in mature NCs further destabilizes them (broken hexagon) and facilitates their uncoating and the release of RC DNA into the nucleus (Nuc). Some HBc subunits may remain associated with RC DNA in the nucleus and modulate its conversion to cM-RC DNA (cM) and ultimately to CCC DNA (CCC). In the transfection experiments, plasmid DNAs harboring the HBV genome (HBV plasmid) serves as surrogate CCC DNA to initiate HBV gene expression and replication. See text for details.

To explore the role of potential HBc NTD phosphorylation in viral replication, we focused here on the two highly conserved putative NTD phosphorylation sites, S44 and S49. Results obtained with the N2A mutant suggested that phosphorylation at these two sites were not essential for HBc expression, capsid assembly, pgRNA packaging, DNA synthesis, and secretion of complete virions. On the other hand, the N2E mutant capsid showed a partial defect in RNA packaging. These results thus suggest that these NTD sites should not be phosphorylated (at least not to any significant degree) at the early stage of virus assembly (e.g., RNA packaging). However, N2E preferentially destabilized mature NCs, and increased the efficiency of PF-RC DNA and CCC DNA formation. In contrast, N2A showed reduced CCC DNA formation efficiency. Together, these results suggest a model that these two NTD sites are non-phosphorylated during NC assembly and maturation, but their phosphorylation, following NC maturation, may facilitate the next stage of replication, i.e., NC uncoating during infection or nuclear recycling to allow CCC DNA formation (Fig 11). The role of CDK2, specifically that packaged in the capsids (i.e., the endogenous kinase), in phosphorylating HBc to trigger NC uncoating for CCC DNA formation is supported by the inhibition of CCC DNA formation by two distinct CDK2 inhibitors during HBV infection. Thus, “premature” phosphorylation of these NTD sites would be detrimental to viral replication as it would impair pgRNA packaging. On the other hand, HBc phosphorylation following NC maturation would facilitate viral replication by stimulating NC uncoating for subsequent CCC DNA formation. Consistent with the proposed role of NC phosphorylation in regulating HBV NC uncoating, a previous report suggested a role of a putative CDK1-like phosphorylation site in the duck hepatitis B virus (DHBV) core protein (DHBc) NTD in DHBV replication, particularly in NC uncoating [65].

An endogenous kinase activity in HBV capsids was discovered nearly four decades ago [66]. However, both the identify and the function of the endogenous kinase remain to be clearly clarified. We have recently reported the major endogenous kinase is likely to be CDK2 (or a close relative) [45]. What is the purpose, if any, of the packaged host kinase for the virus remains unknown. Phosphorylation of an interior capsid site(s), such as the S44 and S49 studied here, may explain the need to package a host kinase during assembly and virus production, since a host cell kinase may not have access to the capsid interior, or it may not be expressed (or active) in the quiescent hepatocyte that HBV encounters during infection. Both S44 and S49 are also within the conserved SP motifs in the HBc NTD, which are well-known CDK consensus substrate sites. How the timing of NTD phosphorylation may be regulated is not yet clear. However, the removal of the viral envelope layer, upon fusion of the host and viral membrane during viral entry, would allow access by the packaged kinase inside the NC to cytoplasmic ATP, which could trigger the endogenous kinase activity to initiate phosphorylation of the HBc NTD sites (as well as CTD sites, see below). Envelopment of mature NCs during virion secretion will shut off the endogenous kinase by depriving it of ATP. How phosphorylation at the NTD sites is suppressed during the early stages of replication, as we propose here, is more difficult to understand. One possibility is that these NTD residues are sequestered and inaccessible to the cellular kinase(s) until NC maturation-triggered structural changes render them exposed to the endogenous kinase. The very low (if any) levels of phosphorylation of the 7E and 9E mutants, which are unable to package pgRNA or synthesize viral DNA [30, 43], during EKR in vitro is also consistent with a previous report showing that the isolated HBc NTD sequence (from 1–117) was apparently not phosphorylated in vitro by a capsid-associated kinase (CAK) [48], suggesting that HBc phosphorylation outside the CTD (i.e., in the NTD and/or linker) may be tightly regulated and requires additional factors (such as NC maturation).

Our Phos-tag gel analysis suggested that the 9E mutant core protein could indeed still be phosphorylated in human cells (either in the NTD and/or linker), as evidenced by its slower mobility on the Phos-tag gel. Also, the clear upshift of 9E as compared to the lack of upshift of 7E, together with the enhanced phosphorylation of N2E compared with the WT HBc, is consistent with the notion that HBc phosphorylation could be stimulated by phosphorylation at the two NTD sites (S44 and S49). We attempted to further verify the mobility upshift of 9E on the Phos-tag gel was indeed due to phosphorylation by pretreating the core proteins with the alkaline phosphatase before the Phos-tag gel analysis. As shown in S3 Fig, the phosphatase treatment indeed clearly showed that the slower mobility of the WT, 2A, 2E, 3A, and 3E proteins was due to their phosphorylation as phosphatase pretreatment clearly increased their mobilities on the gel. However, it is also clear that dephosphorylation (even after overnight treatment) was incomplete, even with the HBc proteins translated in the rabbit reticulocyte lysate, which were dephosphorylated prior to their assembly that can be triggered by dephosphorylation [30]. The incomplete dephosphorylation suggests that some of the phosphorylation sites might be sequestered from the phosphatase either by HBc subunit conformation or by the assembly of the capsid particle. This incomplete dephosphorylation makes the interpretation difficult of the results of phosphatase treatment of the 7A, 7E, 9A, 9E mutants, the mobilities of which were apparently unaffected by the phosphatase treatment (S3 Fig).

Also, N2E and 3E (and possibly 7E and 9E), i.e., the phosphor-mimetic mutations, all showed increased levels of phosphorylation than the corresponding N2A and 3A (and 7A/9A) mutations, suggesting that phosphorylation at one site of the capsid can facilitate phosphorylation elsewhere on the capsid, i.e., capsid phosphorylation may be “processive” and “cooperative.” Using phospho-selective anti-CTD antibodies, we could verify that N2E indeed increased CTD phosphorylation. Also, we and others have recently shown that phosphorylation at the three SP sites in the CTD, as mimicked by the 3E mutation, can stimulate phosphorylation at one or more of the remaining (non-SP) CTD sites [30, 46], a result confirmed here by EKR in vitro using isolated capsids as well as Phos-tag gel analysis of HBc proteins extracted from cells. This phosphorylation cascade or “processive”/“cooperative” phosphorylation of HBc will lead to transient hyper-phosphorylation of mature NCs, leading to rapid and efficient NC uncoating by amplifying the destabilizing effect of phosphorylation at any one site. On the other hand, the transient (and potentially sub-stoichiometric, i.e., less than 240 copies HBc per capsid) nature of phosphorylation at the HBc NTD (and possibly the linker region as well) renders it difficult to detect these events directly.

Our Phos-tag gel conditions were able to resolve more than a dozen of different phospho-HBc species, which should prove useful for further elucidating the dynamics of HBc phosphorylation at different stages of HBV replication. On the other hand, based on the low state of phosphorylation of the 7A and 7E mutants revealed by our Phos-tag gel analysis here and previous metabolic labeling experiments [23, 30, 36, 45], it seems unlikely that HBc is highly phosphorylated outside the CTD. The multiple species of phosphor-HBc resolved on the gel (over a dozen) does not necessarily mean that there are 12 or more sites of phosphorylation on HBc; rather, different combinations of phosphorylation at the seven CTD and the two NTD sites (and possibly other sites) may lead to differential mobility on the gel even if they have the same number of sites of phosphorylation, as suggested by a recent study [50]. It also remains possible that conformational effects induced by HBc phosphorylation (or mutation) might also contribute to the mobility shift on the Phos-tag gel.

How N2E (or NTD phosphorylation) may stimulate NC uncoating remains to be elucidated. It will increase local negative change at the monomer-monomer interface (Fig 1) within the HBc dimer–the building block of HBV capsids [15], thus weakening the dimer and the capsid. Furthermore, the progressive and cooperative phosphorylation of HBc triggered by NTD S44/S49 phosphorylation should lead to a dramatic increase in the overall negative charge of the NC interior, given the interior of localization of the NTD sites as well as most of CTDs [15]. This overall increase of NC interior negative charge may be a major driving force in triggering mature NC uncoating/disassembly. As the total levels of capsids, which represent mostly empty capsids [5, 30, 58], were not significantly affected by N2E or N2A, we can further suggest that NTD phosphorylation at these sites by itself is insufficient to trigger capsid disassembly but that the internal negative charges contributed by the partially double stranded RC DNA in mature NCs also play an important role in triggering uncoating, presumably due to electrostatic repulsion between the RC DNA negative charges and the negative charges from the hyperphosphorylated HBc proteins. The rigidity of the partially double-stranded RC DNA with mature NCs may also exert internal pressure to facilitate their disassembly [67]. In addition, we have previously shown that HBc NTD mutations such as I126A and L60A can destabilize mature NCs [26], suggesting NTD structural changes modulate NC uncoating. Thus, phosphorylation of HBc (at NTD and/or CTD) may also facilitate mature NC uncoating by altering the NC structure beyond their effects on the NC charge state. Our results here obtained using HBc NTD phosphorylation mutants, together with our previous results showing that the related DHBc CTD phosphor-mimetic mutants also preferentially destabilize mature DHBV NCs [40] are consistent with the notion that whereas NC dephosphorylation during maturation is required to facilitate RC DNA synthesis and stabilization of mature NCs, subsequent rephosphorylation of mature NCs facilitates disassembly (uncoating) to release the mature RC DNA for CCC DNA formation (Fig 11). Although genetic analyses of HBc CTD phosphorylation mutants so far has not been able to provide clear support for a functional role of HBc dephosphorylation in NC maturation due to the pleiotropic effects of such mutations on multiple stages of HBV replication [31, 36], our recent biochemical analysis indicates that NC dephosphorylation similarly occurs during maturation in HBV [43], as in DHBV [39]. Thus, the phosphorylation-dephosphorylation-rephosphorylation cycle during pgRNA packaging, reverse transcription, and uncoating, as depicted in Fig 11, likely applies to both HBV as well as DHBV. Whereas our results here provide support for a role of HBc NTD phosphorylation in HBV CCC DNA formation by facilitating NC uncoating, the role of the HBc CTD, including its phosphorylation state, in CCC DNA formation is difficult to test directly by mutagenesis studies as substitutions of the CTD sites of phosphorylation to mimic either phosphorylation or dephosphorylation block pgRNA packaging or RC DNA synthesis (see Introduction above), steps preceding and prerequisite for NC uncoating and CCC DNA formation.

Little is currently understood about the mechanisms of HBV NC disassembly/uncoating. We have previously shown that mature NCs are less stable than immature NCs, which likely primes mature NCs selectively for disassembly (uncoating) to release its interior content, i.e., RC DNA, for conversion to CCC DNA in the host cell nucleus [27]. We have also demonstrated that the HBc NTD can play an important role in the preferential destabilization of mature NCs [26]. In addition, the HBc CTD state of phosphorylation can affect capsid stability [24, 40, 41, 60, 68]. In particular, DHBc mutations that mimic constitutive phosphorylation and prevent CTD dephosphorylation allow the accumulation of immature NCs and dramatically destabilize mature NCs [40]. Furthermore, host specific factors are also involved in regulating NC uncoating. One mouse hepatocyte cell line, in contrast to normal mouse hepatocytes and other mouse hepatocyte cell lines, can support HBV CCC DNA formation and become susceptible to HBV infection upon HBV receptor reconstitution [63, 69, 70]. The ability to support HBV CCC DNA formation and HBV infection is correlated with enhanced destabilization of mature NCs in this mouse hepatocyte cell line [63]. Therefore, it is likely that a failure of mouse hepatocyte to support HBV NC uncoating underlies its inability to support HBV CCC DNA formation, which represents a major intracellular block to HBV infection and a major determinant of HBV species tropism.

Another potential mechanism that can account for the effect of the NTD mutations on HBV CCC DNA levels is their possible effect on nuclear import of mature NCs. Although all known nuclear localization signals (NLSs) of HBc have been mapped to the CTD, not NTD, the CTD state of phosphorylation is known to affect HBc nuclear localization [23, 71]. The putative NTD phosphorylation identified here may affect nuclear import of mature NCs via its effect on CTD phosphorylation. NC uncoating could also enhance the exposure of the NLSs on CTD, which may be mostly sequestered inside the capsid. Furthermore, N2E enhanced the levels of cM-RC DNA, a likely intermediate during the CCC DNA formation. In addition to its stimulatory effect on NC uncoating, which can lead to increased production of cM-RC DNA from the released RC DNA, this result suggest that N2E might also affect the further conversion of cM-RC DNA to CCC DNA, which would also lead to accumulation of cM-RC DNA even more than CCC DNA, as we observed here (Fig 11). HBc NTD phosphorylation thus, may also play a more direct role in regulating the pathway of CCC DNA formation, specifically affecting the conversion of cM-RC to CCC DNA, e.g., by affecting the subnuclear localization or compartmentalization of RC DNA or cM-RC DNA. This result further implies that following NC uncoating, some HBc subunits remain associated with RC DNA during at least some stage(s) of its conversion to CCC DNA in the nucleus.

The fact that the N2A mutant still formed PF-RC DNA and CCC DNA indicates that the mature NC could uncoat without phosphorylation at these two NTD sites albeit at reduced levels. This may be especially true in human hepatoma cells in culture which may not fully recapitulate the intracellular environment that HBV finds itself in human hepatocytes in the liver. The host cell environment that HBV encounters upon entry into permissive cells may thus influence the requirement for HBc NTD phosphorylation to induce uncoating, e.g., increased levels of host kinase activity may induce increased phosphorylation at other HBc sites to compensate for the loss of S44/49 phosphorylation. The apparently variable effects of N2A on PF-RC DNA and CCC DNA we observed here in transfected hepatoma cells are consistent with the notion that yet-to-be defined host cell factors can modulate the requirement for NTD phosphorylation in triggering mature NC uncoating for CCC DNA formation. On the other hand, infection of primary human hepatocytes (the PXB cells), which do not divide in culture and more closely mimic the human hepatocytes in vivo than hepatoma cells, and of HepG2-NTCP cells that were arrested in growth by DMSO pretreatment, showed that the N2A mutant virus consistently made less CCC DNA compared to WT, thus supporting an important role of S44/49 phosphorylation in CCC DNA formation during HBV infection of authentic host cells. Moreover, as the non-cycling PXB cells likely had little to no CDK2 activity, the inhibition of CCC DNA formation during HBV infection of these cells further suggests that the endogenous CDK2 packaged into the HBV capsids was targeted by the inhibitors during infection to block NC phosphorylation, uncoating, and CCC DNA formation, thus implicating the endogenous CDK2 in these critical processes.

Other than the cytokine type I interferon, which has both direct antiviral and immunomodulatory activities, all currently approved therapeutic agents for chronic hepatitis B target the viral RT DNA synthesis activity [72, 73]. Despite being very effective in suppressing HBV replication, they fail to completely eradicate HBV. This is mainly due to the persistence of viral CCC DNA inside the infected hepatocytes. Small molecule compounds targeting the HBc NTD are being pursued actively as potential therapeutics [74, 75]. Some of these compounds can inhibit capsid assembly or pgRNA packaging. Furthermore, consistent with the pleiotropic role of HBc in multiple stages of viral replication beyond capsid assembly and pgRNA packaging, particularly in controlling NC uncoating, HBc-targeted compounds have recently been shown to affect CCC DNA formation, possibly via modulating NC disassembly/uncoating [74–81]. Our results here further suggest that HBc-targeted agents may affect CCC DNA formation beyond NC uncoating by influencing events inside the nucleus. Therefore, a better understanding of HBV NC assembly/disassembly and CCC DNA formation will likely inform ongoing efforts for the development of novel HBc-directed anti-HBV therapy targeting these processes.

## Supporting information

S1 Fig Packaged pgRNA or core DNA detected by (-) strand or (+) strand RNA probe separately. The HBV genomic construct expressing the WT, N2A, or N2E mutant HBc was transfected into HepG2 cells. Following transfection, entecavir (ETV, 200 nM) was added to the culture medium and maintained for five days. Thereafter, cytoplasmic lysate was prepared from the transfected cells using 1% NP-40 and resolved on a 1% agarose gel. Upon transfer of the resolved capsids onto nitrocellulose membrane, the packaged pgRNA was detected using a ^32^P-labeled anti-sense riboprobe (A, lanes 1–6). The packaged DNA was detected using a ^32^P-labeled sense riboprobe and phosphorimaging scan (B, lanes 7–12). Subsequently, capsids were detected on the same membrane by using the mouse monoclonal anti-HBc antibody T2221 and chemiluminescence.
(TIF)

S2 Fig EKR in the presence of CDK2 inhibitors. The WT HBc expression construct were transfected into HepG2 cells. Cytoplasmic lysate was prepared from the transfected cells using 1% NP-40 five days after transfection. The lysate was treated with 0.5 ug/ul proteinase K at 37°C for one hr before EKR in the presence of [γ-^32^P]ATP. The CDK2 inhibitor roscovitine (Ros), K03861 or CDK2 inhibitor III (CDK2i III) was added at the beginning of EKR at the indicated concentrations. The reaction products were resolved on an agarose gel. Upon transfer of the resolved capsids onto nitrocellulose membrane, radiolabeled (phosphorylated) capsid levels resulting from the EKR were measured using phosphorimaging (Top). Total capsid levels were detected on the same membrane by using the mouse monoclonal anti-HBc antibody T2221 and chemiluminescence (Bottom). Ca, capsid. Phosphorylation efficiency during EKR was measured by normalizing the levels of labeled capsids to total capsids, with that from the WT capsid set to 1.0.
(TIF)

S3 Fig Phosphatase pretreatment of HBc proteins before Phos-tag gel analysis. The WT and mutant HBc proteins were translated in the rabbit reticulocyte lysate in the presence of ^35^S-methionine as described before [30]. All samples were resolved by Phos-tag SDS-PAGE. Where indicated, the translation reactions were incubated overnight at 37°C in 1x NEB restriction digestion buffer 3 alone (lanes 1, 3, 5, 7, 9, 11, 13, 15 and 17) or with the calf intestine alkaline phosphatase (CIAP) (lanes 2, 4, 6, 8, 10, 12, 14, 16 and 18) [30] before resolution on the gel. ^35^S-labeled HBc proteins were detected using phosphorimaging. C-P, phosphorylated HBc; C-deP, dephosphorylated (non-phosphorylated) HBc. Note the partially dephosphorylated N2E species (lane 6) migrating above the respective species of WT (lane 2) and 2A (lane 4) HBc.
(TIF)
