# Supplementary figures and images for: Role of Hepatitis B virus capsid phosphorylation in nucleocapsid disassembly and covalently closed circular DNA formation
Source: PLoS Pathog. 2020 Mar 30;16(3):e1008459. doi: 10.1371/journal.ppat.1008459 (PMC7145273; doi:10.1371/journal.ppat.1008459)

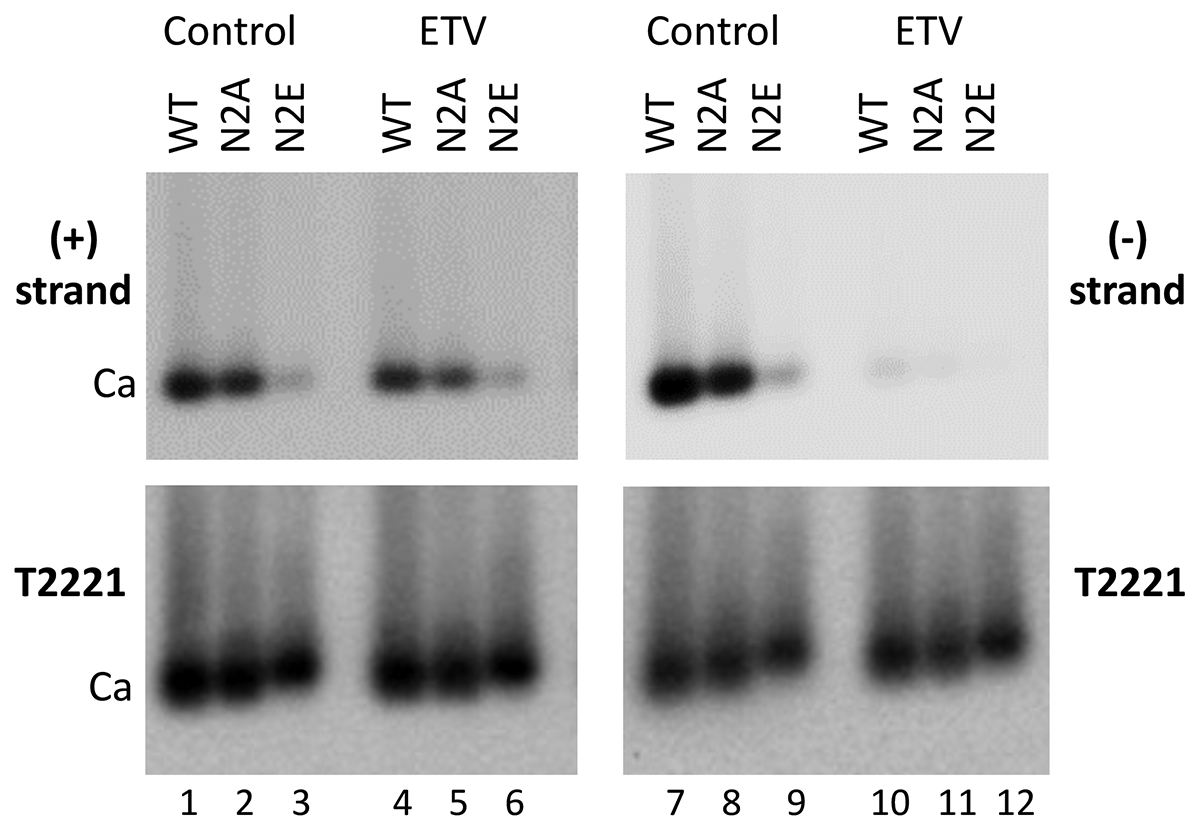

Supplement: S1 Fig — The HBV genomic construct expressing the WT, N2A, or N2E mutant HBc was transfected into HepG2 cells. Following transfection, entecavir (ETV, 200 nM) was added to the culture medium and maintained for five days. Thereafter, cytoplasmic lysate was prepared from the transfected cells using 1% NP-40 and resolved on a 1% agarose gel. Upon transfer of the resolved capsids onto nitrocellulose membrane, the packaged pgRNA was detected using a 32P-labeled anti-sense riboprobe (A, lanes 1–6). The packaged DNA was detected using a 32P-labeled sense riboprobe and phosphorimaging scan (B, lanes 7–12). Subsequently, capsids were detected on the same membrane by using the mouse monoclonal anti-HBc antibody T2221 and chemiluminescence. (TIF) [file ppat.1008459.s001.tif]

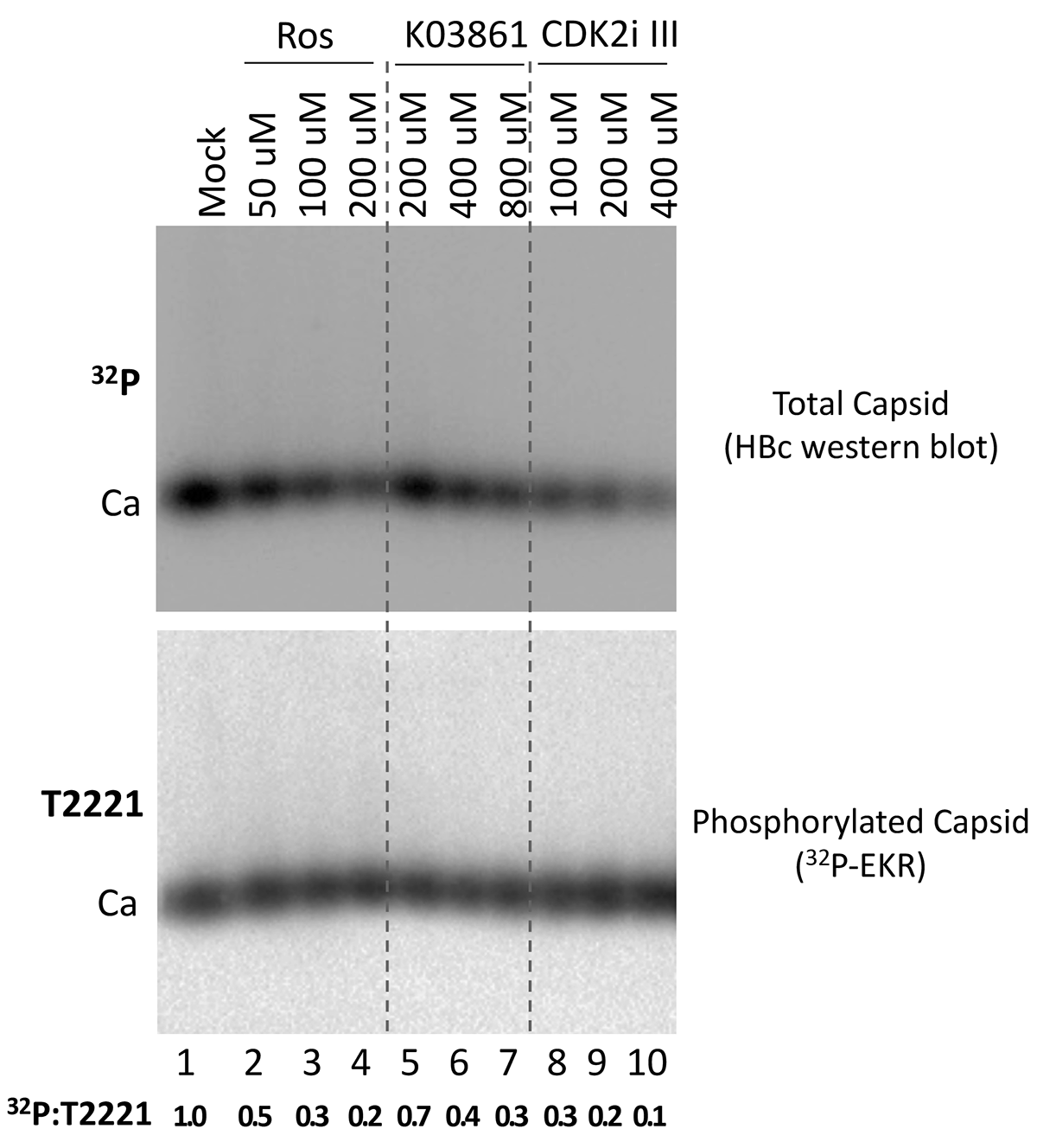

Supplement: S2 Fig — The WT HBc expression construct were transfected into HepG2 cells. Cytoplasmic lysate was prepared from the transfected cells using 1% NP-40 five days after transfection. The lysate was treated with 0.5 ug/ul proteinase K at 37°C for one hr before EKR in the presence of [γ-32P]ATP. The CDK2 inhibitor roscovitine (Ros), K03861 or CDK2 inhibitor III (CDK2i III) was added at the beginning of EKR at the indicated concentrations. The reaction products were resolved on an agarose gel. Upon transfer of the resolved capsids onto nitrocellulose membrane, radiolabeled (phosphorylated) capsid levels resulting from the EKR were measured using phosphorimaging (Top). Total capsid levels were detected on the same membrane by using the mouse monoclonal anti-HBc antibody T2221 and chemiluminescence (Bottom). Ca, capsid. Phosphorylation efficiency during EKR was measured by normalizing the levels of labeled capsids to total capsids, with that from the WT capsid set to 1.0. (TIF) [file ppat.1008459.s002.tif]

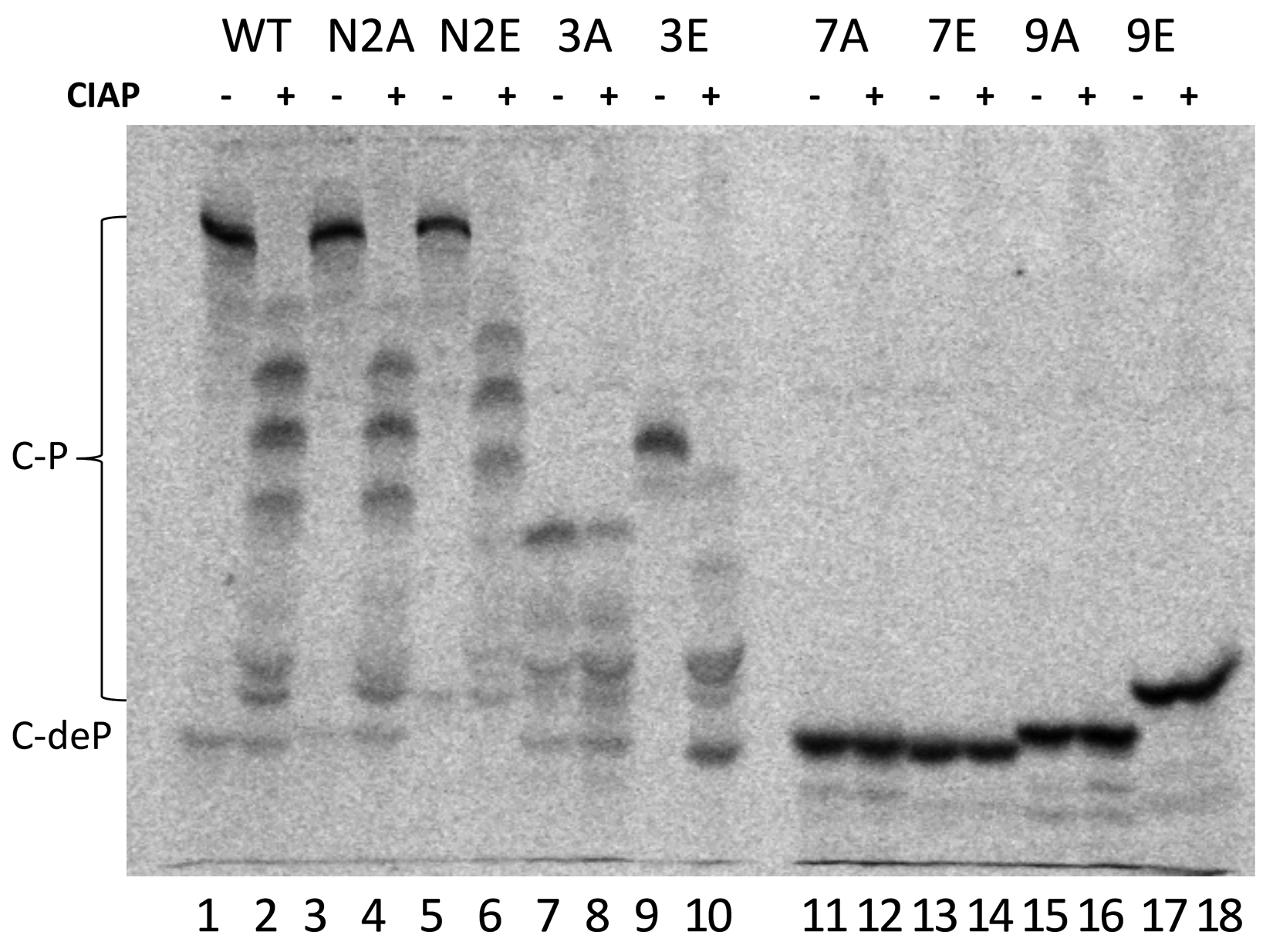

Supplement: S3 Fig — The WT and mutant HBc proteins were translated in the rabbit reticulocyte lysate in the presence of 35S-methionine as described before [30]. All samples were resolved by Phos-tag SDS-PAGE. Where indicated, the translation reactions were incubated overnight at 37°C in 1x NEB restriction digestion buffer 3 alone (lanes 1, 3, 5, 7, 9, 11, 13, 15 and 17) or with the calf intestine alkaline phosphatase (CIAP) (lanes 2, 4, 6, 8, 10, 12, 14, 16 and 18) [30] before resolution on the gel. 35S-labeled HBc proteins were detected using phosphorimaging. C-P, phosphorylated HBc; C-deP, dephosphorylated (non-phosphorylated) HBc. Note the partially dephosphorylated N2E species (lane 6) migrating above the respective species of WT (lane 2) and 2A (lane 4) HBc. (TIF) [file ppat.1008459.s003.tif]
